# A naturally occurring 22-amino acid fragment of human hemoglobin A inhibits autophagy and HIV-1

**DOI:** 10.1007/s00018-024-05447-1

**Published:** 2024-09-17

**Authors:** Dennis Freisem, Armando A. Rodriguez-Alfonso, Jan Lawrenz, Zhixuan Zhou, Thomas Monecke, Nico Preising, Sascha Endres, Sebastian Wiese, Ludger Ständker, Seah-Ling Kuan, Dietmar R. Thal, Tanja Weil, Dierk Niessing, Holger Barth, Frank Kirchhoff, Mirja Harms, Jan Münch, Konstantin M. J. Sparrer

**Affiliations:** 1 Institute of Molecular Virology, Ulm University Medical Center, Meyerhofstraße 1, 89081 Ulm, Germany; 2 Core Facility Functional Peptidomics, Ulm University, Meyerhofstraße 4, 89081 Ulm, Germany; 3 Core Unit Mass Spectrometry and Proteomics, Ulm University Medical Center, Albert-Einstein-Allee 11, 89081 Ulm, Germany; 4 Institute of Experimental and Clinical Pharmacology, Toxicology and Pharmacology of Natural Products, Ulm University Medical Center, Albert-Einstein-Allee 11, 89081 Ulm, Germany; 5 Max Planck Institute for Polymer Research, Ackermannweg 10, 55128 Mainz, Germany; 6 Institute of Pharmaceutical Biotechnology, Ulm University, James-Franck-Ring N27, 89081 Ulm, Germany; 7 Laboratory of Neuropathology, Institute of Pathology, Center for Clinical Research at the University of Ulm, 89081 Ulm, Germany; 8 Present Address: Laboratory of Neuropathology, Department of Imaging and Pathology, Leuven Brain Institute, KU Leuven, Louvain, Belgium

**Keywords:** Hemoglobin, Innate immunity, HIV, Autophagy

## Abstract

Autophagy is an evolutionarily ancient catabolic pathway and has recently emerged as an integral part of the innate immune system. While the core machinery of autophagy is well defined, the physiological regulation of autophagy is less understood. Here, we identify a C-terminal fragment of human hemoglobin A (HBA1, amino acids 111–132) in human bone marrow as a fast-acting non-inflammatory inhibitor of autophagy initiation. It is proteolytically released from full-length HBA1 by cathepsin E, trypsin or pepsin. Biochemical characterization revealed that HBA1(111–132) has an in vitro stability of 52 min in human plasma and adopts a flexible monomeric conformation in solution. Structure–activity relationship studies revealed that the C-terminal 13 amino acids of HBA1(120–132) are sufficient to inhibit autophagy, two charged amino acids (D127, K128) mediate solubility, and two serines (S125, S132) are required for function. Successful viruses like human immunodeficiency virus 1 (HIV-1) evolved strategies to subvert autophagy for virion production. Our results show that HBA1(120–132) reduced virus yields of lab-adapted and primary HIV-1. Summarizing, our data identifies naturally occurring HBA1(111–132) as a physiological, non-inflammatory antagonist of autophagy. Optimized derivatives of HBA1(111–132) may offer perspectives to restrict autophagy-dependent viruses.

**Supplementary Information:**

The online version contains supplementary material available at 10.1007/s00018-024-05447-1.

## Introduction

Autophagy is an evolutionarily ancient catabolic mechanism essential for cellular homeostasis, mediating the turnover of cytoplasmic content, including proteins and organelles [1]. As part of the cell intrinsic immune defenses, autophagy mediates the degradation of viruses and viral components [2, 3]. Induction of autophagy by cellular stress or viral infections is governed by a set of kinases including AMPK and ULK1, which promote autophagy induction, while mTOR and CK2 negatively regulate ULK1 activity [4–7]. Upon induction of autophagy, double-layered isolation membranes are formed, derived from Golgi- or ER membranes, that engulf cytoplasmic cargo [3, 7] in an unspecific (bulk degradation) or highly specific manner (selective autophagy). During selective autophagy, cargo, for example obsolete organelles, protein aggregates or viral components, are recruited by dedicated autophagy receptors such as SQSTM1/p62 [8, 9]. The budding autophagy vesicles, called autophagosomes, are decorated with a lipidated, membrane-bound version of the protein LC3B. LC3B which is processed from its cytoplasmic version (LC3B-I) to its autophagosome-bound version (LC3B-II), is frequently used as an autophagy marker marker of autophagy [10]. Eventually, the autophagosome closes and fuses with a lysosome to form autophagolysosomes. The cargo and inner membrane are subsequently degraded, and the resulting nutrients, such as amino acids, are recycled back into the cytoplasm. While the core machinery of autophagy has been extensively studied in the past decades [7, 11], the physiological regulation of autophagy, especially between cells, remains incompletely understood.

Successful viruses such as the human immunodeficiency virus 1 (HIV-1) have evolved strategies to counteract or even exploit autophagy to establish an infection [3, 12, 13]. Accessory proteins disturb the autophagic flux, thereby preventing targeting and degradation of incoming virions or viral proteins [3, 12, 14–16], as well as promoting its virion production at later stages of infection [3, 17]. Thus, while activation of autophagy restricts HIV-1 at early stages, at later time points the production of infectious virions can be reduced by inhibiting autophagy [2, 3]. Current approaches to therapeutically manipulate autophagy rely on broad-acting compounds, such as Rapamycin and Chloroquine [3]. However, this rather unspecific activation or inhibition of autophagy is not well tolerated by patients. As such, the clinical application of autophagy modulation is largely limited to cancer therapy [18, 19]. In addition to chemical compounds, pro-inflammatory cytokines such as interferon γ (IFN-γ), tumor necrosis factor α (TNF-α), or interleukin-1 (IL-1) have previously been recognized as inducers of autophagy [20–22]. However, in addition to inducing autophagy, they also mediate detrimental inflammation. Endogenous peptides that regulate autophagy independently of inflammation are currently unknown.

Hemoglobin (HB) is the second most abundant protein after type I collagen in the human body, with blood concentrations reaching 120–180 g/l (18–28 mM) in adults [23]. It is a tetramer consisting of two alpha and two beta chains expressed from the HB subunit alpha-1 (HBA1) and HB subunit beta-1 (HBB1) genes, respectively [24]. In addition to its essential role in binding and transporting oxygen in red blood cells [24], HBA1 and HBB1 are precursors of several bioactive peptides generated by endogenous proteases [25–28]. Fragments of HB can be detected in various tissues and body fluids, such as the spleen, uterine secretions, the placenta, and menstrual blood [25–27, 29, 30]. These fragments were reported to exhibit extra-erythrocytic functions, ranging from analgesic to hemopoietic roles [31]. Emerging evidence indicates that fragments of HB contribute to innate immune defense as anti-microbial or immunomodulatory peptides [25–27, 29]. For example, a C-terminal fragment of human HBB1 found in placenta was reported to inhibit herpes simplex virus (HSV) 2 [30]. A fibril forming C-terminal part of HBA1 restricts replication of measles and herpes viruses (HSV-1, HSV-2, HCMV) [27]. Furthermore, N-terminal fragments of both HBA1 and HBB1 were reported to promote activation, differentiation, and recovery of T lymphocytes in mice [29]. Thus, it is likely that numerous functions of HB derived fragments remain unexplored.

Here, we identified a 22-amino acid fragment representing amino acids 111–132 of HBA1 as a physiological autophagy inhibitor isolated from human bone marrow by chromatographic fractionation that restricts HIV-1 virion production in a dose-dependent manner. Mechanistically, HBA1(111–132) reduces autophagic flux by rapidly preventing lipidation and processing of LC3B. Structure–activity relationship (SAR) studies revealed that a conserved C-terminal 13-mer fragment (120–132) is sufficient for autophagy inhibition and two serine residues (S125, S132) are required for its function.

## Materials and methods

### Expression plasmids

Transmitted founder viruses HIV-1 M subtype A 191845 [32] and HIV-1 M subtype B pTHRO.c [33] were kindly provided by the Beatrice Hahn and John Kappes Laboratories. pRRL.cPPT.SF-eGFP.pre was described previously [34, 35]. pRSV-rev (0.25 µg, Addgene plasmid # 12,253, a gift from Didier Trono [36]) and pMDLg/pRRE (3 µg, Addgene plasmid # 12,251, a gift from Didier Trono [36]) were received from Addgene. Vesicular stomatitis virus glycoprotein (VSV-G, 0.5 µg) was described previously [37].

### Cell culture

Cell lines (HEK293T, HeLa, THP-1) were commercially obtained from ATCC (#CRL-3216, #CLL-2, #TIB-202). These cells also represent the origin of the corresponding autophagy reporter cell lines as described in [37]. A549-Dual and THP1-Dual cells were purchased from InvivoGen (#a549d-nfis, #thpd-nfis). TZM-bl cells were provided and authenticated by the NIH AIDS Reagent Program, Division AIDS, NIAID, NIH from Dr. John C. Kappes, Dr. Xiaoyun Wu and Tranzyme Inc. HEK293T(-GL), HeLa(-GL), TZM-bl and A549-Dual cells were cultivated in Dulbecco’s Modified Eagle Medium (DMEM, Gibco) supplemented with 10% (v/v) fetal bovine serum (FBS, Gibco), 6.5 µg/ml Gentamycin (Gibco) and 2 mM L-glutamine (PAN-Biotech). THP-1(-GL) and THP-1 Dual cells were cultivated in Roswell Park Memorial Institute (RPMI, Gibco) 1640 medium supplemented with 10% (v/v) fetal bovine serum (FBS, Gibco), 6.5 µg/ml Gentamycin (Gibco) and 2 mM L-glutamine (PAN-Biotech). Cells were regularly tested for mycoplasma contamination and used only if negative.

### Generation of peptide libraries

Human femur bones and human lungs were obtained by Prof. Dr. Dietmar R. Thal (Institute of Pathology University Medical Center Ulm, Germany). The bone marrow or lung tissue were washed with 1.25 L of 3 M HAc (Sigma). The extract was ultracentrifuged (38,400 g Beckmann Coulter), and the supernatant was filtered using filters with a pore size ranging from 20, 8, 5, 3, 1.2 to 0.45 μm (Sartorius). The clear supernatant (2 L) was diluted with water and applied to an ultrafiltration (Pall) step (cut-off: 30 kDa), resulting in a volume of 2.5 L. The ultrafiltrate was loaded onto a 25 × 3 cm reversed-phase column (Sepax Poly RP-300, 10 μm, Sepax Technologies) at a flow-rate of 40 ml/min. Here, the flow-through (D) was collected. The column was washed with 2 column volumes of 95% A (0.1% TFA (Merck) in water) and 5% B (0.1% TFA in acetonitrile (ACN)), resulting in the pre-fraction (V). Then, the main fractions were eluted, applying a gradient at 40 ml/min from 95% A mixed with 5% B to 100% B in 55 min. After the gradient, the column was washed with 2 column volumes of 95% A and 5% B, which was also collected (fraction: N). Fractions were aliquoted (1% each) in deep well microtiter tubes (Brand). The aliquoted samples were then lyophilized and stored at –20 °C. The remaining bulks were stored at –80 °C. In total, 21 peptide containing fractions were collected (fractions 10–30) from purification and three pre- or postprocessing fractions (D, V, N). The generation of the human placenta library was described previously [30].

### Peptide sequencing by LC–MS/MS

The sample was reduced with 5 mM DTT for 20 min at RT, carbamidomethylated with 50 mM iodoacetamide for 20 min at 37 °C, and quenched with 10 mM DTT. A 15 µl-aliquot was analyzed by a nanoLC-Orbitrap Elite Hybrid mass spectrometry system (Thermo Fisher Scientific) as previously described [5]. Database searches were performed using PEAKs XPro (PEAKs studio 10.6). For peptide identification, MS/MS spectra were correlated with the UniProt human reference proteome set, www.uniprot.org. Carbamidomethylated cysteine was considered as a fixed modification along with oxidation (M) and deamidation (NQ) as variable modifications. False discovery rates were set to 1% on peptide level.

### Peptide synthesis and quality controls

All peptides were synthesized as previously described in detail [27, 38]. Automated resin-based solid phase peptide synthesis was performed at a 0.10 mmol scale by standard Fmoc solid phase peptide synthesis using a microwave synthesizer (Liberty blue; CEM). Wang preloaded resin with serine was washed and soaked in N, N-Dimethylformamide (DMF). Once the synthesis was completed, the peptide was cleaved with 95% (v/v) trifluoroacetic acid (TFA), 2.5% (v/v) triisopropylsilane (TIS) and 2.5% (v/v) water for one hour. The peptide was precipitated in cold diethyl ether (DEE) and subsequently washed three times with DEE and then dried in vacuum. All peptides were purified using reversed phase preparative high-performance liquid chromatography (HPLC; Waters) (Supplementary Fig. S7) in an ACN/water gradient under acidic conditions on a Phenomenex C18 Luna column (5 mm pore size, 100 Å particle size, 250 mm length, 21.2 mm diameter). Subsequently, the peptide was lyophilized on a freeze dryer (Labconco). The peptide mass was verified by liquid chromatography mass spectroscopy (LCMS; Waters).

### Autophagy quantification via flow cytometry

A detailed protocol for quantification of the number of autophagosomes has previously been published [37]. In brief, HEK293T, HeLa, and THP-1 cell lines stably expressing eGFP-LC3B (labelled as -GL respectively) were used. After cells were treated for 4 h with respective substance/fraction, cells were harvested by removing the medium and adding 0.05% trypsin (PAN Biotech). Afterwards cells were washed once with PBS and permeabilized with 0.05% (w/v) Saponin (Sigma-Aldrich) in PBS. Non-membrane bound eGFP-LC3B was washed out using PBS. Cells were fixed in 4% (w/v) paraformaldehyde in PBS. Quantification of the membrane associated eGFP-LC3B was performed using flow cytometry FACS Canto II, BD Biosciences. The mean eGFP-LC3B fluorescence intensity of the water control was then subtracted from each sample.

### Whole-cell lysate

Cells were washed once with PBS and then harvested in transmembrane lysis buffer (50 mM HEPES pH 7.4, 150 mM NaCl, 1% Triton X-100, 5 mM ethylenediaminetetraacetic acid (EDTA)), supplemented with 1:500 protease inhibitor. After incubation for 5–10 min on ice, they were vortexed at maximum speed and centrifuged (20,000 g, 4 °C, 20 min). The cleared lysates were then taken and the total protein concentration was measured using the Pierce BCA Protein Assay Kit (ThermoFisher). The protein concentration of the lysates was then adjusted and lysates were stored at –20 °C until further use.

### SDS-PAGE and immunoblotting

Whole-cell lysates were reduced using a 6 × Protein Sample Loading buffer containing 15% β mercaptoethanol (final dilution of 1x) and denatured at 95 °C for 5 min. Samples were loaded on a 12% acrylamide (Carl Roth) Bis–Tris gel for 120 min at 90 V. The transfer on an Immobilon-FL PVDF membrane (Merck) was performed at 30 V for 30 min. Non-specific binding sites were blocked using 1% Casein (ThermoFisher) in PBS for 30 min. Membranes were then incubated with the primary antibody at 4 °C overnight. After washing three times with PBS + 0.2% tween (Sigma-Aldrich), the membrane was incubated in secondary antibodies labelled with an infrared dye which were diluted in 0.05% Casein in PBS. Visualization and quantification were performed using Image Studio lite (Ver 5.0).

### Cell viability assays

To assess the metabolic activity after treatment, cells were incubated with 500 µg/ml 3-(4,5-Dimethylthiazol-2-yl)-2,5-diphenyltetrazoliumbromide in PBS and incubated for 3 h at 37 °C. The supernatant was discarded and dimethyl sulfoxide mixed with ethanol in equal quantities was added. Absorption was measured at 490 nm using a Vmax Kinetic ELISA microplate reader (Molecular Devices). Background was corrected for absorbance at 650 nm. For membrane integrity analysis, the Fixable Viability Dye eFluor 780 (ThermoFisher) was used according to the manufacturer’s instruction. Afterwards, cells were harvested using 0.05% trypsin (PAN Biotech) and fixed in 4% (w/v) paraformaldehyde in PBS and analyzed via flow cytometry (FACS Canto II, BD Biosciences).

### Autophagy quantification by immunofluorescence

HeLa GL cells were grown on coverslips (VWR) and treated for 4 h with respective substance. Afterwards, cells were fixed with 4% (w/v) PFA (Santa Cruz) for 20 min and permeabilized as well as blocked with 0.5% (v/v) Triton-X-100 (Sigma-Aldrich) and 5% (v/v) fetal bovine serum (Gibco) PBS for 1 h, both at RT. Cells were then mounted on microscope slides (VWR) in 4′,6-diamidino-2-phenylindole (DAPI, VWR)-containing Mowiol mounting medium (Mowiol 4–88 10% (w/v, Carl Roth), glycerol 25% (w/v, Sigma-Aldrich), H_2_O 25% (v/v), 0.2 M Tris HCl pH 8.5 50% (v/v, AppliChem GmbH, VWR), DABCO 2.5% (w/v, Carl Roth)) to co-stain nuclei. After acquiring ≥ 30 randomly selected cells using a Zeiss LSM 710 confocal microscope, a custom ImageJ (Version 1.54j) macro was used to analysis the total area of the cytoplasmic eGFP-LC3B puncta as previously described [37].

### In vitro stability determination

A sample (0.5 ml) of human plasma was spiked with 8.7 µM HBA1(111–132) (P69905) and incubated at 37 °C. Aliquots (50 µl) were separated at 0, 15, 30, 60, 120, 180, 240, 360, and 1440 min, respectively, and mixed with 250 µl 0.1% TFA in acetonitrile at –20 °C. The mixture was centrifuged at 13,000 rpm for 30 s, and 150 µl of the supernatant was mixed with 750 µl 10% acetic acid in ice. The samples were analyzed by an Axima Confidence MALDI-TOF MS (Shimadzu) in linear mode using exactly the same measurement conditions for all samples spotted on a 384-well plate. Wells were coated with 1 µl of 5 mg/ml CHCA previously dissolved in matrix diluent (Shimadzu), and the solvent was allowed to evaporate. Then, each sample (0.5 µl), previously mixed with matrix (0.5 µl), was applied onto the dry pre-coated well, and the solvent was allowed to evaporate. Laser shots were automatically done following a regular circular raster of a diameter of 2000 µm and spacing of 200 µm on each well; 100 profiles were acquired per sample, and 20 shots were accumulated per profile. An accelerating voltage of 20 kV was applied to the ion source. Three experiments were performed, and the measurements of each sample were done in triplicate (81 measurements in total). The measurement and MS data processing (peak area calculation) were controlled by MALDI-MS Application Shimadzu Biotech Launchpad 2.9.8.1 (Shimadzu). Half-lives were calculated by GraphPad Prism 9.3.1 (GraphPad Software, LLC). EPI-X4, which has a known half-life, was added as an internal quality control to ensure accurate quantification [39].

### Quantification of inflammatory signaling

Type I IFN: A549-Dual and THP1-Dual (InvivoGen) cells were seeded and treated with respective substance for 24 h at 37 °C. After incubation, 25 µl supernatant was taken and transferred to a white Nunc F-bottom 96-well plate. The plate was put into an Orion II Microplate Reader (Berthold) which automatically added 50 µl of 20 µM coelenterazine (PJK biotech) and after a 2 s delay measured the relative light units per second (RLU/s). NF-ĸB pathway: For quantification of the NF-ĸB pathway signaling again A549-Dual and THP1-Dual cells were used. After 24 h of treatment, 20 µl of supernatant was transferred to a F-bottom 96-well plate and mixed with the alkaline phosphatase blue microwell substrate (Sigma-Aldrich), according to instruction. After 10 min of incubation the plate was analyzed at a wavelength between 490 and 650 nm, using a Vmax kinetic microplate reader (Molecular Devices).

### Localization by flow cytometry

HeLa cells were seeded and treated with V5-tagged-HBA1(111–132) (GKPIPNPLLGLDSTGGGGSGGGGSAAHLPAEFTPAVHASLDKFLAS) for 30 min. Afterwards, cells were harvested using TrypLE Express (Gibco) and fixed with 4% (w/v) PFA (Santa Cruz) for 10 min. Subsequent staining was done using a V5-tag rabbit monoclonal antibody (Cell Signaling Cat#13202S Lot: 7) 1:200, either permeabilized with or unpermeabilized without 0.1% (v/v) Tween (Sigma-Aldrich) as well as blocked with 1% (v/v) fetal bovine serum (Gibco) PBS for 30 min. Cells were washed in PBS and then incubated with the secondary antibody anti-rabbit Alexa 488 (ThermoFisher, Cat#A-11008 Lot: 2382186) 1:200 for 30 min. After washing with PBS, cells were taken up in 1% (v/v) fetal bovine serum (Gibco) in PBS and analyzed via flow cytometry (FACS Canto II, BD Biosciences).

### Protease digestion experiment

For the digestion of human HB, 100 µg (1.56 nM) of purified human HB (Sigma H7379) was digested for 2 h at 37 °C with different recombinant or purified human proteases in the respective buffer. HB was digested with chymase (Sigma C8118, in 0.05 M Tris–HCl, 0.26 M NaCl, pH 8.0), cathepsin D (Sigma C8696, in 0.2 M citrate buffer, pH 5.0), cathepsin G (Invitrogen RP77525, in 0.2 M citrate buffer, pH 5.0), cathepsin L (R&D Systems 952-Cy, in 50 mM MES, 5 mM DTT, 1 mM EDTA, Brij35 0.005%, pH 6.0), cathepsin E (R&D Systems 1294-AS, in 0.2 M citrate buffer, pH 5.0), pepsin (Sigma 77,160, in 2 mM NaAcetate, pH 2.0), trypsin (Sigma T0303, in 0.1 M Tris–HCl, 10 mM CaCl2, pH 8.0) and napsin A (R&D Systems 8489-NA, in 0.2 M NaCl, 0.1 M NaOAc, pH 3.6). All proteases were used in a 1:100 molar ratio (15 pM) of protease to HB in the digestion mix. For the SDS-Page, samples were incubated with Protein Loading Buffer (LiCOR 928-40004) and reducing agent TCEP before heating for 10 min at 70 °C. 4 µg of digested HB was loaded per lane on a NuPAGE 4–12% Bis–Tris Protein Gel (Thermo Fisher). Following this, the gel was fixed with 50% MeOH/ 7% acetic acid for 15 min and washed 3 × 5 min with ultrapure water. Subsequent coomassie staining was performed for 1 h with GelCode Blue Reagent (Thermo Fisher 24,590). The gel was destained using ultrapure water until the background appeared clear and then imaged using a gel imaging system (ChemDoc MP, Bio-Rad Laboratories).

### MS for detection of HBA1 fragments in digest

The digested samples (cathD, cathE, cathG, cathL, pepsin, trypsin, chymase, napsin A) were subjected to mass spectrometry analysis for the identification of HBA1(111–132) and HBA1(120–132) fragments. A 15 µl aliquot was analyzed by a nanoLC-Orbitrap Elite Hybrid mass spectrometry system (Thermo Fisher Scientific) as previously described [5]. Database search was performed using MaxQuant Ver. 1.6.3.4 [40]. Employing the built-in Andromeda search engine [41], MS/MS spectra were correlated with the UniProt human HBA protein as a reference (Uniprot entry P69905, HBA_human). Using unspecific digest, parent mass error tolerance and fragment mass error tolerance were set at 15 ppm and 0.5 Da, respectively. Carbamidomethylated cysteine was considered as a fixed modification along with oxidation (M), and acetylated protein N-termini as variable modifications. False discovery rates were set to 0.01 at both, peptide and protein level.

### Circular dichroism

Circular dichroism spectroscopy was recorded on a JASCO J-1500 spectrophotometer at 25 °C using a 0.1 cm path length quartz cuvette from Hellma Analytical. Data points were collected at a resolution of 0.2 nm, an integration time of 2 s and a scanning speed of 5 nm min-1. Each spectrum was the result of three accumulated independent scans. Phosphate buffer solution (10 mM, pH = 7.4) was employed to solubilize the samples. To achieve the optimum high tension (HT) voltage in the measurements, the concentration of the samples in the measurements was diluted to 130 µM for HBA1(120–132), 54 µM for HBA1(111–132), and 2.1 µM for full-length human HB. Background subtraction was performed using the solvent. Secondary structure estimation (SSE) for the samples was conducted using the CD Multivariate SSE program included in the JASCO spectrometer.

### Molar mass determination of peptides in solution by multi-angle light scattering (MALS)

Multi-angle light scattering coupled to size exclusion chromatography (SEC-MALS) was used to determine the absolute molar mass of the peptides to specify their oligomeric state in solution. For this, 100 µl 5 mg/ml HBA1(111–132) or 10 mg/ml HBA1(120–132) in 1 × PBS was applied onto an analytical Superdex Increase S30 10/300 column (Cytiva), equilibrated in 1 × PBS at a flow rate of 0.5 ml/min. Peptides eluting from the column were detected using a setup consisting of a DAWN8 light scattering detector directly coupled to an Optilab dRI detector (Wyatt Technology). Data were analyzed and molar masses were calculated using the ASTRA 8.0 software. The molar masses were calculated to be 2535 Da ± 1.6% for HBA1(111–132) (theoretical monomer 2293 Da) and 1465 Da ± 1.3% for HBA1(120–132) (theoretical monomer 1355 Da).

### Determination of peptide solubility using a precipitation turbidity assay

To rank the solubility of the different peptides (HBA1(120–132), scrambled HBA1(120–132, SALADKSLAPVHF), scrambled HBA1(120–132, FSLKDLVASAAPH), HBA1(120–132)S125A, HBA1(120–132)S132A, HBA1(120–132)S125A + S132A, HBA1(120–132)D127A, HBA1(120–132)K128A, HBA1(120–132)D127A + K128A) the turbidity of solutions containing 1 mg/ml peptide in 1 × PBS was measured. 100 µl peptide solution was transferred to transparent 96-well F-bottom microplates (Greiner) and immediately processed. Before the measurement, solutions were briefly mixed by double-orbital shaking for 60 s at 240 rpm. Measurements were done at 22 °C in triplicates using a TECAN Spark plate reader at a wavelength of 330 nm and a bandwidth of 3.5 nm. Values were corrected against 1 × PBS and averaged.

### HIV-1 production

To produce HIV-1, 135,000 HEK293T cells per well were seeded in a 24-well F-bottom plate in medium. The day after, cells were treated 15 min prior to transfection with HBA1(120–132) (1 mg/mL–2 mg/mL) and Bafilomycin A1 (125 nM). Cells were transfected using 3 µL of TransIT-LT1 (Mirus, MIR2306) per 1 µg of DNA with the proviral cDNA constructs. After 16 h the medium was changed and cells were treated with indicated substance as well as 24 h post transfection. Virus was harvested 48 h post transfection and transferred to TZM-bl cells.

### TZM-bl reporter assay

Relative infectious virus yields of HIV-1 containing supernatant were quantified by seeding 10,000 TZM-bl cells per well in a 96-well F-bottom plate in 100 µL medium. After 24 h, cells were then infected with HIV-1 containing supernatant and incubated for another 48 h. The supernatant was then discarded and 40 µl Gal-Screen β-galactosidase Reporter Gene Assay substrate (Invitrogen, T1028) diluted 1:4 in PBS (Gibco, 14190144) was added to the cells. After 30 min of incubation at RT, 35 µl substrate was transferred to a white 96-well F-bottom plate and the β-galactosidase signal was measured for a period of 0.5 s using an Orion II Microplate Reader (Berthold).

### ELISA p24

To determine HIV-1 p24 amounts in cell culture supernatant an in-house ELISA was used. In brief, 96-well microplates MaxiSorp (Sigma) were incubated overnight with 0.5 mg/ml anti-HIV-1 p24 (EXBIO #11-CM006-BULK) in a wet chamber at RT. After three times washing with PBS-T (PBS + 0.05% Tween20), plates were incubated at 37 °C with blocking solution (PBS + 10% (v/v) FCS) for 2 h. After subsequent washing, plates were loaded with 100 µl of virus supernatant dilutions lysed using 1% (v/v) of Triton X-100 and a serial dilution of HIV-1 p24 protein (Abcam #ab43037) as standard and incubated in a wet chamber at RT, overnight. After washing, 100 µl of a polyclonal rabbit antiserum against p24 antigen (Eurogentec, 1:1000 in PBS-T with 10% (v/v) FCS) was added in each well, incubating for 1 h at 37 °C. After washing, 100 µl of a goat anti-rabbit HRP-coupled antibody (Dianova#111-035-008, 1:2000) was added to each well for 1 h at 37 °C. Eventually, plates were washed again and 100 µl SureBlue TMB 1-Component Microwell Peroxidase Substrate (Medac #52-00-04) was added. After 20 min shaking at 450 rpm RT, the reaction was stopped using 0.5 M H2So4 (100 µl/well). The colorimetric shift was determined via measurement at 450 nm and 650 nm with the Thermo Max microplate reader (Molecular devices). Results were compared to the respective standard curve.

### Quantification and statistical analysis

Statistical analyses were performed using GraphPad PRISM 9. P-values were calculated using a two-tailed Student’s t test with Welch’s correction or One-way ANOVA for multiple comparisons (Mann–Whitney test). Unless specified otherwise, data are shown as the mean of at least three biological replicates ± SEM. Significant differences are indicated as: *p < 0.05; **p < 0.01; ***p < 0.001. Insignificant differences are not indicated.

## Results

### Autophagy-inhibiting fractions of a human bone marrow library contain fragments of hemoglobin A

**Fig. 1:**
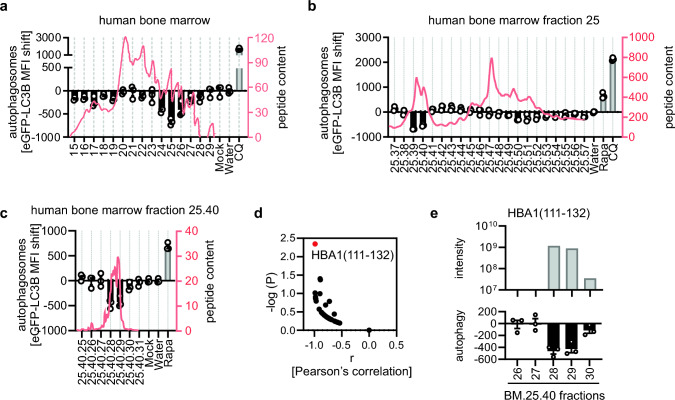
Identification of HBA1(111–132) in autophagy inhibiting fractions of a human bone marrow library. **a–c** Autophagosome levels in HeLa reporter cells stably expressing GFP-LC3B (HeLa GL) as assessed by flow cytometry 4 h post treatment with fractions of a human bone marrow peptide library (**a**), subfractions of human bone marrow fraction 25 (**b**), subfractions of human bone marrow library 25.40 (**c**). Fraction numbers are indicated. Water control set to 0. Bars represent mean of n = 3 ± SEM. CQ, Chloroquine (1 µM). Rapa, Rapamycin (1 µM). Absorbance at 214 nm is displayed as red line (right y-axis). **d** Pearson’s correlation between activity (autophagosome levels) and abundance (peptide intensity) of the hundred most abundant hits (x-axis). Significance of the correlation, -log(p) (y-axis). Red dot indicates HBA1(111–132). **e** Autophagy modulating activity in BM.25.40.26–30 as in (**c**) and mass spectrometry intensity for HBA1(111–132) in indicated fraction

To discover endogenous regulators of autophagy, we analyzed a human peptide library derived from bone marrow (BM) [42, 43]. In brief, marrow was removed from 1.5 kg human femur bones and homogenized in acetic acid. The homogenate was filtered to contain only peptides with a molecular mass below 30 kDa [27, 42]. The BM library was initially separated on a reversed-phase column. To analyze the autophagy-modulating properties of individual peptide fractions, we used a high-throughput flow cytometry-based autophagosome quantification system [37]. A hallmark of autophagy is the transition from soluble LC3B-I to autophagosome-associated LC3B-II. In GFP-LC3B expressing HeLa cells (HeLa GL), soluble GFP-LC3B is washed out by mild permeabilization and the remaining membrane-bound GFP-LC3B is quantified by FACS as a proxy for autophagosome numbers [37]. Changes in autophagosome content are visualized as deviations from the background (i.e. carrier) control. Analysis of 15 peptide-containing fractions (absorption at 214 nm above 0) of the BM library revealed significant downregulation of autophagosomes in fraction BM.24–26, peaking in fraction BM.25 (Fig. 1a). Subsequent chromatographic separation of fraction BM.25 on a reversed-phase column, revealed that subfractions BM.25.39/40 inhibited autophagy (Fig. 1b). Further purification identified subfraction BM.25.40.28 as bioactive (Fig. 1c). To identify the active peptide(s) we subjected fractions BM.25.40.26 to BM.25.40.30 to analysis by mass spectrometry (Supplementary data 1). Peptides with a molecular mass from ~ 750 Da to 5360 Da and a length from 7 to 57 amino acids were found in the fractions (Supplementary Fig. 1a). In the most active fraction, BM.25.40.28, 337 unique peptides, with HBB1(90–111, intensity 3.01E + 09) and HBA1(111–132, intensity 1.18E + 09) being the most abundant (Supplementary data 1, Supplementary Fig. 1b, c) were found. To pinpoint the active peptide from the 337 peptides identified, we correlated the abundance of the contained peptides with autophagy activity over BM.25.40.26 to BM.25.40.30 (Fig. 1d). These analyses revealed that HBA1 (111–132, sequence: AAHLPAEFTPAVHASLDKFLAS) exhibited the strongest and most significant correlation with the activity of the analyzed fractions (Fig. 1d, e).

Taken together, our analysis identified HBA1(111–132) in autophagosome inhibiting fractions of a human bone marrow-derived peptide library.

### Chemically synthesized HBA1(111–132) reduces autophagic flux and is stable in human plasma

**Fig. 2:**
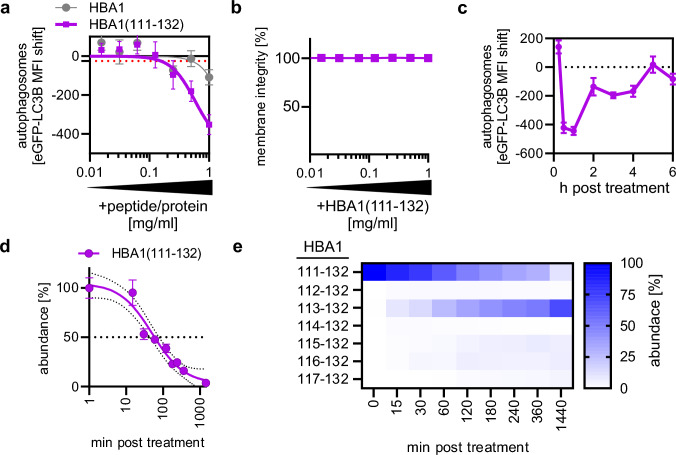
HBA1(111–132) reduces autophagic flux. **a** Autophagosome levels in HeLa GL cells assessed by flow cytometry 4 h post treatment with full-length HBA1 and synthesized HBA1(111–132) in a concentration range of 1 mg/ml–7.8 µg/ml. Red dotted line indicates the standard error. Dots represent mean of n = 3 ± SEM. **b** Membrane integrity of HeLa GL cells after treatment with varying concentrations (1 mg/ml–7.8 µg/ml) of HBA1(111–132) for 4 h, assessed by flow cytometry. Bars represent mean of n = 3 ± SEM. **c** Autophagosome levels in HeLa GL cells assessed by flow cytometry at indicated time points (15 min–6 h) post treatment with synthesized HBA1(111–132) at a concentration of 1 mg/ml. Dots represent mean of n = 3 ± SEM. **d** Abundance of synthesized HBA1(111–132) after incubation in human plasma at different time points as monitored by mass spectrometry. Small dotted lines indicate the 95% confidence interval. Big dotted line indicates 50% abundance. n = 9 (3 technical replicates of 3 biological replicates) ± SEM. **e** Heatmap of fragments originating from synthesized HBA1(111–132) after incubation with human plasma. Abundance % represents the intensity ratio of the respective fragment in regard to the total intensities of all fragments at this time point as assessed via mass spectrometry.

HBA1(111–132) was chemically synthesized and tested to understand whether it regulates autophagy (Supplementary data 2). Treatment of autophagy reporter HeLa GL cells with serial dilutions of HBA1(111–132, 1 mg/ml to 15.6 µg/ml) reduced the amount of autophagosomes in a dose-dependent manner (Fig. 2a). The minimum effective concentration (MEC) at which the peptide caused an effect below the standard error (Fig. 2a, red dotted line) was 0.156 mg/ml and the calculated inhibitory concentration 50% (IC_50_) was 0.59 mg/ml. Of note, full-length HBA1 did not impact autophagosome levels (Fig. 2a). HBA1(111–132) did not affect cellular metabolic activity (Supplementary Fig. 2a) and membrane integrity (Fig. 2b), suggesting it is not cytotoxic. Autophagosome levels were also reduced in THP-1 monocytes and HEK293T autophagy reporter cells upon treatment with HBA1(111–132) in a dose-dependent manner, suggesting that the effect is independent of the cell line (Supplementary Fig. 2b). Time-course analysis revealed that HBA1(111–132) reduced autophagosome levels almost immediately after application, reaching a maximum at around 30 min post treatment (Fig. 2c). Afterwards, the impact waned, reaching baseline at around 5 h post treatment (Fig. 2c). Human plasma contains proteases responsible for the turnover of proteins and peptides [44]. The stability of HBA1(111–132) was assessed in human plasma ex vivo using mass spectrometry, showing that HBA1(111–132) has an in vitro half-life of ~ 52.4 min (Fig. 2d). Analysis of the degradation products of HBA1(111–132) indicates digestion from the N-terminus at every second amino acid (Fig. 2e, Supplementary Fig. 2c). Thus, the truncated HBA1(113–132) is generated already within the first 15 min (above 5% of total HBA1 content), peaking at >140 min post plasma treatment, replacing HBA1(111–132) as the dominant HBA1 fragment. This is followed by the delayed appearance of HBA1(115–132) above 5% of the total HBA1 content at 180 min post plasma treatment.

Collectively, biochemical characterization of synthetic HBA1(111–132) revealed that it reduces autophagosomes at concentrations > 0.156 mg/ml, is non-cytotoxic up to 1 mg/ml, fast-acting and has a 50% stability of ~ 52 min in human plasma. Active concentrations of HBA1(111–132) are thus ~ 50-fold lower than achieved by proteolytic processing of the highly abundant precursor HBA1 in blood [25, 27].

### HBA1(111–132) adopts a flexible and monomeric conformation in solution

**Fig. 3:**
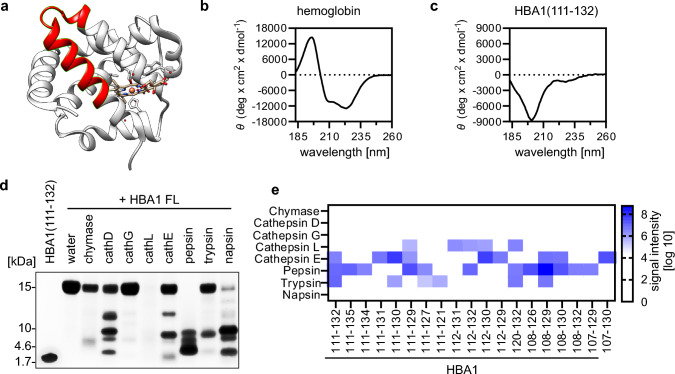
Biochemical analyses and origin of HBA1(111–132). **a** Visualization of full-length hemoglobin with amino acid sequence 111–132 highlighted in red. Structure derived from PDB: 6BB5. **b–c** Circular dichroism spectrum of full-length recombinant HBA1 (**b**) and HBA1(111–132) (**c**) in 10 mM PBS pH = 7.4 at 25 °C. **d** Exemplary SDS-PAGE of digested full-length hemoglobin with indicated proteases. Stained using Coomassie Brilliant Blue. HBA1 FL, Hemoglobin A full-length. **e** Identification of HBA1 fragments from mass spectrometry analysis of gel section at expected height of HBA1(111–132) in (**d**). Color code represents the signal intensity as analyzed by label-free mass spectrometry. Cathepsin (cath)

HBA1(111–132) is derived from the C-terminal alpha helical loop of full-length HBA1 (Fig. 3a, structural visualization based on PDB: 6BB5 [45]). Circular dichroism analysis of recombinant full-length HB in solution confirmed that it is mainly composed of alpha-helical structures (Fig. 3b). However, synthetic HBA1(111–132) appears unstructured in solution, as revealed by circular dichroism analyses (Fig. 3c). To determine whether HBA1(111–132) is monomeric or oligomeric in solution, we performed size-exclusion chromatography coupled to multiangle light scattering (SEC-MALS). The results showed that the peptide exclusively forms monomers in solution at the expected molar mass of ~ 2545 Dalton (Supplementary Fig. 3a).

These data indicate that HBA1(111–132) adopts a flexible, monomeric conformation in solution despite being derived from an alpha-helical part of full-length HBA1.

### HBA1(111–132) is generated by proteolytic digestion of full-length HBA1

Previous data indicate that fragments of HBA1 are typically generated by proteolytic digestions at low pH [27, 30]. To analyze which proteases are responsible for releasing HBA1(111–132), purified full-length HB was digested in vitro with the acidic proteases cathepsin D, G, L, E, pepsin, and napsin A, as well as trypsin and chymase at basic pH. SDS-PAGE and Coomassie Brilliant Blue gel analysis showed that after digestion of the full-length HBA1 (~ 16 kDa) by cathepsin D, E, pepsin and napsin fragments with similar size as HBA1(111–132) (~ 2 kDa) are produced (Fig. 3d). Subsequent mass spectrometry and ESI–MS/MS analysis readily detected HBA1(111–132) after HBA1 digestion by cathepsin E, pepsin and trypsin (Fig. 3e). Of note, several closely related, slightly larger HBA1 fragments were generated by pepsin as well, including HBA1(111–135) and HBA1(111–134). In contrast, other proteases like cathepsin L only led to the release of smaller fragments, such as HBA1(111–129) or HBA1(112–132). Next, we aimed to understand whether HBA1(111–132) is also generated in other tissues besides human bone marrow. Peptidome analyses by mass spectrometry showed that HBA1(111–132) was readily detected in human lung and placenta as well (Supplementary Fig. 3b, Supplementary data 3).

Taken together, our data indicates that HBA1(111–132) is generated from full-length HBA1 by the proteases trypsin, pepsin, and cathepsin E and occurs in human bone marrow, lung and placenta.

### HBA1(111–132) inhibits autophagy initiation independently of pro-inflammatory signaling

**Fig. 4:**
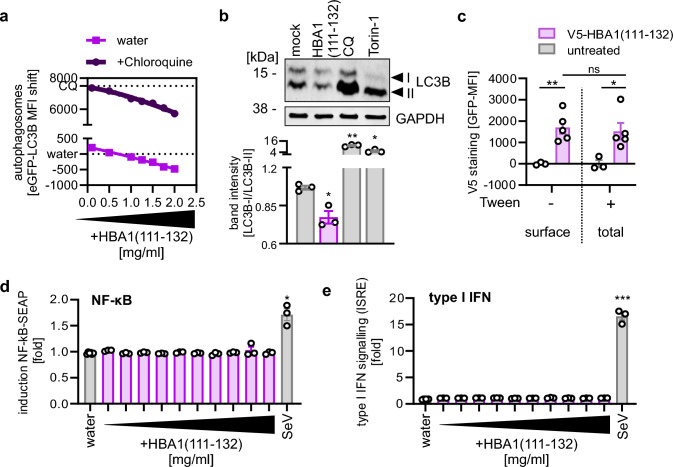
HBA1(111–132) inhibits early autophagy without causing inflammation. **a** Quantification of autophagosome levels in HeLa stably expressing GFP-LC3B reporter cells 4 h post treatment with HBA1(111–132) (2 mg/ml – 0.1 mg/ml) (lower panel) and co-treated with 20 µM Chloroquine (CQ) (upper panel) by flow cytometry. Dots represent mean of n = 3 ± SEM. **b** Exemplary immunoblot analysis of LC3B‑I/-II levels in HeLa cells treated for 4 h with HBA1(111–132) (1 mg/ml), CQ (2 µM) and Torin-1 (1.25 µM). Lower panel: Corresponding quantification of band intensity ratio from LC3B-I to LC3B-II, normalized to mock control. Bars represent mean of n = 3 ± SEM. **c** Flow cytometry analysis of permeabilized (Tween +) and non-permeabilized (Tween-) HeLa cells treated for 30 min with V5-tagged HBA1(111–132) (1 mg/ml) or mock. n = 3–5 ± SEM. **d-e** Quantification of NF-κB driven SEAP expression (**d**) or ISRE driven luciferase expression (**e**) in THP1-Dual cells treated with HBA1(111–132) (1 mg/ml–2 µg/ml). Sendai virus (SeV, MOI 0.03) was used as a positive control. Bars represent mean of n = 3 ± SEM. Student’s t-test with Welch correction or two-way ANOVA (**c**). *p < 0.05; **p < 0.01; ***p < 0.001

To approach the molecular mechanism of HBA1(111–132), we first examined whether HBA1(111–132) reduces autophagic flux or increases autophagosome turnover. To this end, the flux was stopped by adding a saturating concentration of Chloroquine, which prevents fusion of lysosomes and autophagosomes [10]. Thus, if HBA1(111–132) modulates late stages of autophagy, this is masked in the presence of Chloroquine, whereas alterations at early stages of autophagy are enhanced due to the accumulation of autophagosomes over time. A comparative analysis of the dose-dependent impact of HBA1(111–132) on autophagosomes in HeLa GL cells, under both mock- and Chloroquine-treated conditions, suggests that the HBA1 fragment influences the autophagic flux (Fig. 4a). One of the early steps of autophagy is the conversion of cytoplasmic LC3B into membrane-bound phosphatidylethanolamine(PE)-conjugated LC3B-II [10]. Western blot analysis revealed that HBA1(111–132) reduces the relative abundance of LC3B-II to ~ 70% of mock-treated cells (Fig. 4b). Next, we used functional V5-tagged HBA1(111–132) to analyze whether it acts at the cell surface or is internalized (Supplementary Fig. 4a). Flow cytometry revealed that V5-HBA1(111–132) can be detected at similar levels in permeabilized and non-permeabilized HeLa cells suggesting that HBA1 binds to the cell surface and is not internalized (Fig. 4c, Supplementary Fig. 4b). Cytokines known to affect autophagy also induce pro-inflammatory signaling pathways, like IFN or activation of NF-κB [21]. To understand whether HBA1(111–132) regulates autophagy independently of pro-inflammatory signaling, we analyzed the impact of HBA1(111–132) on both pathways using reporter cells encoding luciferases under control of IFN signaling (IFN stimulated response element, ISRE) or NF-κB promotors. These assays showed that the peptide did not alter induction of NF-κB and IFN-stimulated promoters in A549 or THP-1 cells (Fig. 4d, e, Supplementary Fig. 4c, d).

Collectively, these results indicate that HBA1(111–132) binds to the cell surface and downregulates autophagy by interfering with LC3B lipidation independently of pro-inflammatory signaling.

### The C-terminal part of HBA1(111–132) is sufficient for autophagy modulation

**Fig. 5:**
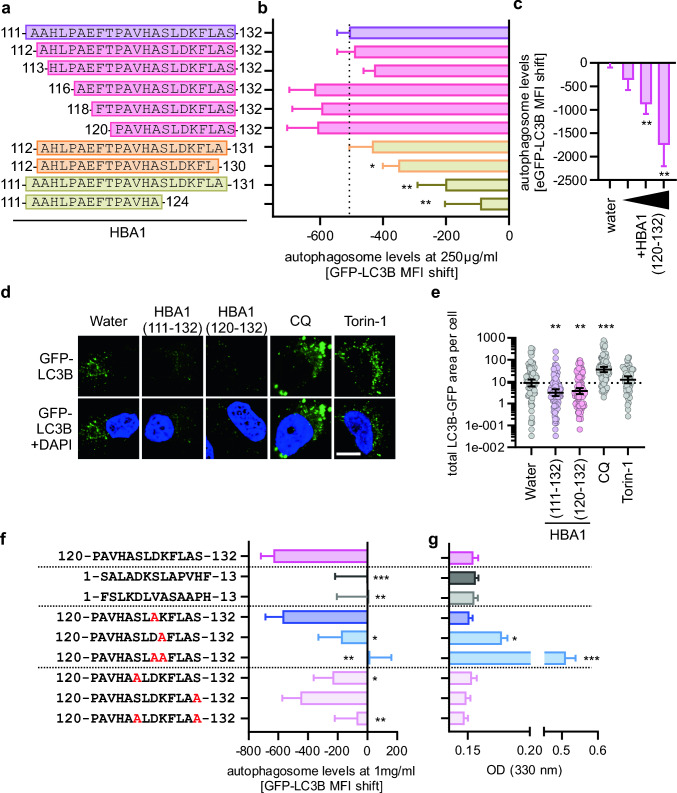
Structure activity relationship studies on HBA1(111–132). **a** Schematic depiction of the synthesized variants of HBA1. **b** Quantification of the autophagosome levels in HeLa GL via flow cytometry 4 h post treatment with indicated HBA1 fragments (**a**) at a concentration of 250 µg/ml. Bars represent mean of n = 6–12 ± SEM. **c** Analysis of autophagosomes in HeLa GL treated with HBA1(120–132) (250 µg/ml, 0.5 mg/ml, 1 mg/ml) for 4 h, assessed by flow cytometry. **d** Representative confocal microscopy images of GFP-LC3B (green) puncta formation in HeLa GL cells, treated for 4 h with HBA1(111–132) (1 mg/ml), HBA1(120‑132) (1 mg/ml), Chloroquine (CQ, 10 µM), Torin-1 (1 µM). Scale bar, 10 µm. DAPI (blue), nuclei. **e** Quantitative analysis of the LC3B-GFP area per cell of the data in (**d**). Lines represent geometric mean with 95% confidence interval, n = 32–110. Dotted line represents the geometric mean of the water sample. **f** Quantification of autophagosome levels in HeLa GL cells after 4 h of treatment with indicated version of HBA1(120–132) and co-treatment of 20 µM Chloroquine (CQ). Bars represent mean of n = 10–24 ± SEM. **g** Assessment of solubility of peptides indicated in (**f**) via optical density measurement of respective mutation of HBA1(120–132) in PBS. Student’s t-test with Welch correction. *p < 0.05; **p < 0.01; ***p < 0.001

To optimize the size of HBA1(111–132), we synthesized truncated peptide variants and assessed their impact on autophagy (Fig. 5a). Functional analysis using flow cytometry of HeLa GL cells upon treatment with 250 µg/ml of N-terminal and C-terminal truncated variants of HBA1(111–132) revealed that deletion of up to 9 amino acids from the N-terminus are well tolerated (Fig. 5b) and HBA1(120–132) is still reducing autophagosome levels in a dose-dependent manner (Fig. 5c). However, deletion of amino acids from the C-terminus markedly reduced activity, with HBA1(111–124) being almost inactive (Fig. 5b). To confirm the activity of HBA1(120–132) using a different readout, analysis of LC3B puncta formation (= autophagosomes) was performed in HeLa GFP-LC3B cells (Fig. 5d). Whereas treatment with the autophagy turnover inhibitor Chloroquine and the autophagy inducer Torin-1 [10] led to the expected increase of autophagosomes, treatment with either HBA1(111–132) or HBA1(120–132) significantly reduced the GFP-LC3B area per cell (Fig. 5e). Similar to HBA1(111–132), HBA1(120–132) adopts a flexible, monomeric conformation in solution, as assessed by circular dichroism and MALS (Supplementary Fig. 5a, b).

Taken together, these assays show that the C-terminal amino acids of HBA1(111–132) are required for autophagy inhibition and identified HBA1(120–132) (PAVHASLDKFLAS) is equally active as HBA1(111–132).

### Residues critical for function of HBA1(120–132)

To determine if the specific amino acid sequence, rather than the composition of HBA1(120–132; PAVHASLDKFLAS) is necessary for autophagy inhibition, we scrambled this sequence. Two scrambled peptides (SALADKSLAPVHF and FSLKDLVASAAPH) were designed by randomizing the original amino acid sequence using peptidenexus.com. Neither scrambled peptide reduced autophagosome levels at 1 mg/ml, suggesting that the amino acid arrangement is crucial for function (Fig. 5f). Overall, HBA1(120–132) is relatively hydrophobic, with an aliphatic index of 105.38 and a grand average of hydropathicity (GRAVY) of 0.477 (Supplementary Fig. 5c). To understand which individual amino acids of HBA1(120–132) are required for autophagy inhibition, we synthesized derivatives with systematically exchanged charged or polar amino acid residues, thereby increasing or decreasing hydrophobicity (Supplementary Fig. 5c). Subsequently, the activity and solubility of the synthesized peptides were analyzed (Fig. 5f, g). The two charged amino acids, a lysine (K127) and an aspartic acid (D128), were both individually exchanged for alanine. While D128A does not alter the function nor the solubility of the peptide, K127A disrupted both, the function to inhibit autophagy was reduced, and precipitates were detected using a precipitation turbidity assay (Fig. 5f, g). Combined exchange of both charged amino acids to the hydrophobic alanine, made the peptide almost insoluble in water, and consequently non-functional (Fig. 5f, g). Besides its hydrophobic amino acids and the two charged amino acids, HBA1(120–132) also contains two polar serines (S125 and S132). While the exchange of each individual Ser to Ala reduced the activity of the peptide, exchange of both serines combined completely disrupted downregulation of autophagy, although the solubility of the peptide remained similar to HBA1(120–132) (Fig. 5f, g).

Overall, these results indicate that the charged residues of HBA1(120–132) are required for solubility, whereas both S125 and S132 are crucial for function. Future work is required to define the precise role of the serine residues.

### HBA1(120–132) inhibits production of infectious HIV-1

**Fig. 6:**
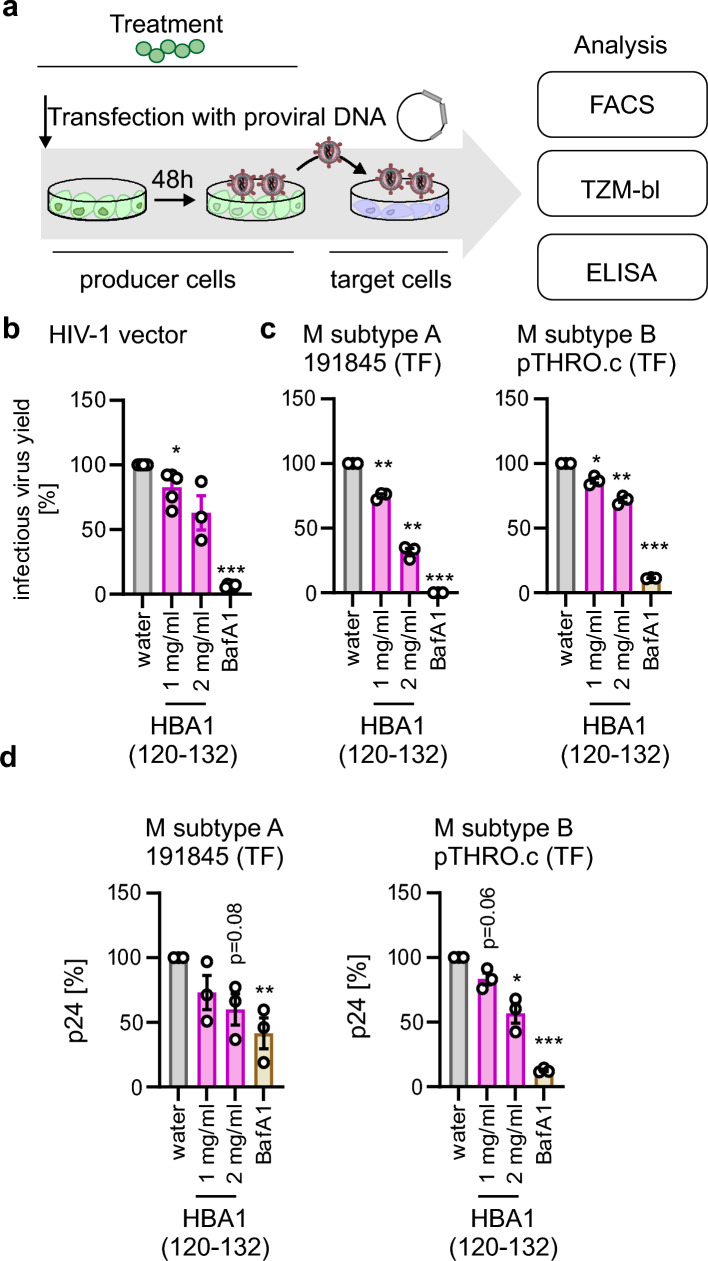
HBA1(120–132) inhibits HIV-1 production. **a** Schematic depiction of virus production and treatment procedure. 15 min before transfection HEK293T cells are treated with HBA1(120–132) or BafA1 (Bafilomycin A1, 250 nM). Cells are then transfected with a pro-viral construct and medium is changed 16 h post transfection. Cells are re-treated with HBA1(120–132) 16 h and 24 h post transfection. After 48 h, supernatant (SN) is transferred to reporter cells (TZM-bl) and 2 days post infection reporter cells are analyzed, either via β-galactosidase assay or flow cytometry. Additionally, the supernatant is analyzed for the presence of p24 capsid protein. **b** Infectious virus yields as assessed by GFP flow cytometry of TZM-bl cells infected with SN from HEK293T transfected with a lentiviral vector, VSV-G, HIV-1 rev and gag/pol and treated as in (**a**). The number of infected (GFP +) cells was normalized to water control. Bars represent the mean of n = 3–6 ± SEM. **c** Infectious virus yields of HEK293T cells transfected with indicated primary transmitted founder (TF) HIV infectious molecular clones as assessed by β-galactosidase assay of TZM-bl 72 h post infection. Treatment with HBA1(120-132) as in (**a**). BafA1 (Bafilomycin A1, 250 nM). Bars represent the mean of n = 3 ± SEM. **d** ELISA quantifying HIV-1 48 h post transfection p24 in the supernatant of HEK293T transfected with indicated pro-viral constructs and treated with different amounts of HBA1(120–132). Bars represent mean of n = 3 ± SEM. **b, c** Student’s t-test with Welch correction. **d** Ratio paired t test. *p < 0.05; **p < 0.01; ***p < 0.001

HIV-1 exploits the autophagic machinery for efficient production of progeny virions [3]. Thus, we analyzed whether inhibition of autophagy by HBA1(120–132) inhibits infectious HIV-1 yields. To this end, we treated HEK293T cells with peptides, transfected them with proviral HIV-1 constructs treated them again and measured infectious virus yield by transferring the supernatants to TZM-bl reporter cells followed by determining the infection rates by either flow cytometry analysis or beta-galactosidase assay [46] (Fig. 6a). Production of an HIV-1 derived lentivirus vector [35] in HEK293T was decreased by more than 50% upon treatment with 2 mg/ml HBA1(120–132) (Fig. 6b, Supplementary Fig. 6a). To understand the impact of HBA1(120–132) on relevant, full-length HIV-1, we used infectious molecular clones of two primary HIV-1 strains (Group M subtype A 191845, Group M subtype B pTHRO.c). Our data shows that infectious virus production of both viruses was inhibited by HBA1(120–132) in a dose-dependent manner, albeit to different degrees (Fig. 6c, Supplementary Fig. 6a). In all cases treatment with the autophagy inhibitor Bafilomycin A1 served as a positive control (Fig. 6b, c). This is associated with a significantly reduced viral protein p24 in the supernatants, suggesting that less virions overall are produced in the presence of HBA1(120–132) (Fig. 6d, Supplementary Fig. 6b).

Taken together, these data indicate that inhibition of autophagy by HBA1(120–132) reduces the infectious virion production of HIV-derived vectors and primary HIV-1.

## Discussion

Here, we identified a naturally occurring fragment of hemoglobin A, HBA1(111–132), as an endogenous inhibitor of autophagy with anti-viral activity. It was isolated from unbiased analysis of autophagy-inhibiting peptide fractions derived from human bone marrow. Mechanistically, our data indicates that HBA1(111–132) inhibits initiation of autophagy with fast kinetics independently of type I interferon or NF-κB signaling. SAR studies revealed that the shorter HBA1(120–132) fragment is as active as HBA1(111–132), suggesting that the activity is conveyed by the C-terminus of the peptide (120–132). Finally, our data suggests that this HBA1 fragment may be part of the innate immune defenses, restricting production of HIV-1 in vitro.

Protease digestion assays showed that HBA1(111–132) is released from full-length HBA1 by trypsin, pepsin, and cathepsin E. This suggests that the peptide could be generated in vivo in the digestive tract (trypsin and pepsin). Curiously, HBA1(111–132) that retains an internal lysine was readily detected after digest of full-length HBA1 with trypsin, which cleaves C-terminal to arginine and lysine[47]. This suggests that longer trypsin digest may lead to a further cleavage of the peptide into even smaller fragments. Degradation of HB is a hallmark of inflammation and infection [27, 48]. Both pepsin and trypsin were previously reported to be involved in inflammatory signaling, however, their exact role is debated [49, 50]. Of note, cathepsin cleavage was reported to be responsible for 75% of the HB digestion at pH 3 and 45% at pH 5 [51]. Additionally, cathepsin E is prominently represented in immune cells [52–55]. Autophagy prevents an exaggerated pro-inflammatory response in the gut, mainly conveyed by the immune responses to commensal or invading microbiome [56, 57]. Thus, it is tempting to speculate that regulation of autophagy by HBA1 fragments may allow to fine-tune the autophagic response to changes in the microbiome, maintaining the delicate gut innate immune homeostasis [58, 59]. Thus, HBA1(111–132) may be specifically generated at sites of microbial infection and contributes to the autophagic response towards pathogens.

HB is expressed at high concentrations in erythroid cells and 150 mg/ml of HB are reached in the blood [60]. In addition, previous reports show that HBA1 is also expressed by nonerythroid cells from human tissues, including lungs, neurons, retina, and endometrium [61]. However, the processing efficiency of HB by proteases and thus in vivo concentrations of HBA1(111–132) in different (inflamed) tissues or fluids are currently unknown. Considering that our data indicates that only as little as 0.6% of the available HB needs to be processed for autophagy inhibiting activity, these concentrations are likely to be reached in vivo.

Our data identified HBA1(111–132) from a bone marrow library and in human lung and placenta tissue. Previous analyses showed that HBA1(111–132) can also be detected in urine and menstrual discharge [25, 26]. In addition, it was elevated in muscle-invasive bladder cancer [25, 26]. Different fragments of HBA1 with immunomodulatory or anti-microbial activity were also previously detected. For example, HBA1(111–142), which forms positively charged fibrils and displays anti-viral activity [27] was detected in fractions derived from menstrual blood with anti-*E. coli* activity [26] and fluid of bacterially-infected wounds [27, 62]. Furthermore, placenta contains HBA1 fragments with anti-bacterial activity [30]. In addition, fragments of HBB1 were also shown to exert innate immune related and anti-microbial properties. HBB(112–147), found from human placenta, shows broad anti-microbial activity against bacteria and viruses including HSV-2 and multi-drug resistant *Pseudomonas aeruginosa* strains [30]. Together with our data, these findings support the general hypothesis that physiologically occurring fragments of HB may contribute to immune homeostasis and defenses.

Our structure activity relationship studies indicate that two serines (S125 and S132) are required for function of HBA1(111–132) (Fig. 5f). At these two positions, single nucleotide polymorphisms altering the amino acid identity, were reported only with a low frequency, but indicating that alterations of these two serines are tolerated [63]: Ser132Ala (Frequency 1.86e-6), Ser125Phe (Frequency 1.24e-6). Of note, synonymous mutations appear more often (Ser132Ser frequency 100-fold higher than Ser132Ala), suggesting that while alterations of these two serines are viable the polymorphisms are more likely to be retained if the amino acid identity is ensured. However, future studies are required to elucidate whether individuals with missense mutations are affected more by specific diseases or infections.

Cytokines or peptides that were previously shown to regulate autophagy also upregulate inflammation [20]. In contrast, HBA1(111–132) did not elevate type I IFN or Nf-κB dependent inflammation. Our mechanistic data indicates that HBA1(111–132) is not internalized. This suggests that engagement of a surface receptor is required to eventually inhibit autophagy. HB scavenger receptors such as haptoglobin, CD163, hemopexin, and the receptor low density lipoprotein receptor-related protein/CD91 have been reported to interact with cell-free HB to prevent potential harmful effects of heme group release [64]. However, it is not known whether these receptors also bind fragments of HBA1. In a patient study, HB levels and the growth hormone IGF-1 have been associated [65]. IGF-1 has been previously implicated in maintaining autophagy levels via the PI3K-mTOR pathway [66–68]. Nonetheless, the receptor of HBA1(111–132) and the involved signaling cascades that eventually regulate autophagy require future studies. However, our data suggests that autophagy-modulating HBA1(111–132) may represent a fundamentally new type of autophagy-modulating peptides.

Besides naturally occurring peptides and cytokines, an artificial peptide that modulates autophagy with high specificity was previously constructed based on a fragment of the autophagic core component beclin-1 fused to a cell penetrating-peptide [69]. This peptide was shown to restrict HIV-1 replication among other viruses in an autophagy-dependent manner after being taken up by the cell [69]. However, this peptide suffers from poor bioavailability due to inefficient intracellular delivery [69].

Our SAR study allowed us to identify a shorter active sequence, residues HBA1(120–132). Furthermore, these studies revealed that two serine residues are crucial for function whereas solubility is mediated by a positive charge. Of note, serine residues can mediate protein–protein interactions but also receive post-translation modifications such as phosphorylations [70]. Furthermore, our data shows that the peptide is degraded from the N-terminus, resulting in an overall in vitro stability half-life of 52 min. Classically, the half-life of cytokines or other immunomodulatory peptides/proteins is very short in human plasma, ensuring termination of the regulation to prevent aberrant or chronic inflammation [71–73]. For example, TNF-α has a half-life of around 18 min [72]. Thus, HBA1(111–132) has a quite long in vitro stability for an immunomodulatory peptide. To improve the stability of HBA1(111–132) for possible therapeutic application, non-natural amino acids or modifications at the N-terminus could be introduced [27, 74]. Of note, the biopolymer nature of peptides makes them ideal starting points for optimization as future immunomodulatory drugs [75].

Our data shows that HBA1(111–132) can limit the production of infectious HIV-1 virions. Autophagy was reported to have a dual role during HIV infection (reviewed in [3]). While autophagy targets and degrades HIV proteins such as the transactivator Tat [76], it also promotes infection as the autophagic machinery was reported to be exploited for virion production [3, 77]. Autophagy often has a dual role during replication of many viruses, suggesting that both activation and inhibition of autophagy may be an effective anti-viral approach [2, 3, 12, 14]. For example, picornaviruses such as encephalomyocarditis virus (EMCV) use autophagic membranes to create replication organelles and support releasing cytoplasmic viruses in a nonlytic manner [78]. Flaviviruses such as Zika virus or Dengue virus have been long known to be promoted by autophagy [79, 80]. Thus, autophagy-inhibiting compounds may restrict the replication of these viruses. However, it has to be considered that HBA1(111–132) shows a moderate autophagy-inhibiting impact at mid micromolar concentrations. Thus, future studies are required to improve the inhibitory effect of the peptide and analyze its impact on other autophagy-dependent viruses.

Currently, therapeutic autophagy modulation plays an important role in anti-cancer therapy [19, 81, 82]. Compounds like Rapamycin, which activates autophagy by inhibiting the mTOR complex, reduces cell proliferation and, thus, tumor progression in vivo [83, 84]. On the other hand, autophagy inhibition e.g. by Chloroquine, which reduces lysosomal pH, sensitizes tumor cells to a variety of conventional anti-cancer treatments, potentiating the therapeutic activity [18, 85, 86]. Several clinical studies have positively evaluated Chloroquine as a possible future anti-cancer drug, slowing cancer progression [87]. However, the currently available drugs, especially autophagy-inhibiting drugs such as Chloroquine, have specificity issues and are thus prone to cause severe adverse effects in patients [88–90]. Considering this, the discovery of HBA1(111–132) as an autophagy modulator may also benefit future cancer therapy.

Collectively, our data identifies HBA1(111–132) as a peptide that inhibits autophagy and reduces HIV-1 virion production. The peptide has been detected in human bone marrow, lung and placenta and may play an important role in shaping the innate immune response against invading pathogens by inhibiting autophagy. Our SAR studies provide a road towards optimization of HBA1(111–132) for future applications against autophagy-dependent viruses or other diseases associated with autophagy dysregulation like cancer or neurodegenerative disorders.

## Supplementary Information

Below is the link to the electronic supplementary material.

Supplementary file1 (PDF 1117 KB)

Supplementary file2 (XLSX 332 KB)

Supplementary file3 (PDF 1341 KB)

Supplementary file4 (XLSX 335 KB)

## Data Availability

The raw data from the mass spectrometry analyses have been uploaded to the MassIVE database (Submission IDs: MSV000095023, MSV000095021, MSV000093288, MSV000095769).
