# Supplementary material for: A naturally occurring 22-amino acid fragment of human hemoglobin A inhibits autophagy and HIV-1
Source: Cell Mol Life Sci. 2024 Sep 17;81(1):409. doi: 10.1007/s00018-024-05447-1 (PMC11408460; doi:10.1007/s00018-024-05447-1)
Supplement: Supplementary file 1 — Supplementary file1 (PDF 1117 KB) [file 18_2024_5447_MOESM1_ESM.pdf]

Supplementary Material:

A naturally occurring 22-amino acid fragment of human hemoglobin A inhibits autophagy and HIV-1

Dennis Freisem[1], Armando A. Rodriguez-Alfonso[2,3], Jan Lawrenz[1], Zhixuan Zhou[5], Thomas Monecke[6], Nico Preising[2], Sascha Endres[3,4], Sebastian Wiese[3], Ludger Ständker[2], Seah-Ling Kuan[5], Dietmar R. Thal[7,8], Tanja Weil[5], Dierk Niessing[6], Holger Barth[4], Frank Kirchhoff[1], Mirja Harms[1], Jan Münch[1] and Konstantin M.J. Sparrer[1,#]

Affiliations:

1 Institute of Molecular Virology
Ulm University Medical Center, Meyerhofstraße 1, 89081 Ulm, Germany
2 Core Facility Functional Peptidomics
Ulm University, Meyerhofstraße 4, 89081 Ulm, Germany
3 Core Unit Mass Spectrometry and Proteomics,
Ulm University Medical Center, Albert-Einstein-Allee 11, 89081 Ulm, Germany
4 Institute of Experimental and Clinical Pharmacology, Toxicology and Pharmacology of Natural Products, Ulm University Medical Center, Ulm, Germany
5 Max Planck Institute for Polymer Research
Ackermannweg 10, 55128 Mainz, Germany
6 Institute of Pharmaceutical Biotechnology, James-Franck-Ring N27
Ulm University, 89081 Ulm, Germany
7 Laboratory of Neuropathology, Institute of Pathology, Center for Clinical Research at the University of Ulm, 89081, Ulm, Germany
8 Laboratory of Neuropathology, Department of Imaging and Pathology, Leuven Brain Institute, KU Leuven, Leuven, Belgium (Current address)

# Correspondence: Konstantin M.J. Sparrer, Konstantin.Sparrer@uni-ulm.de

Supplementary Figures S1-S7:

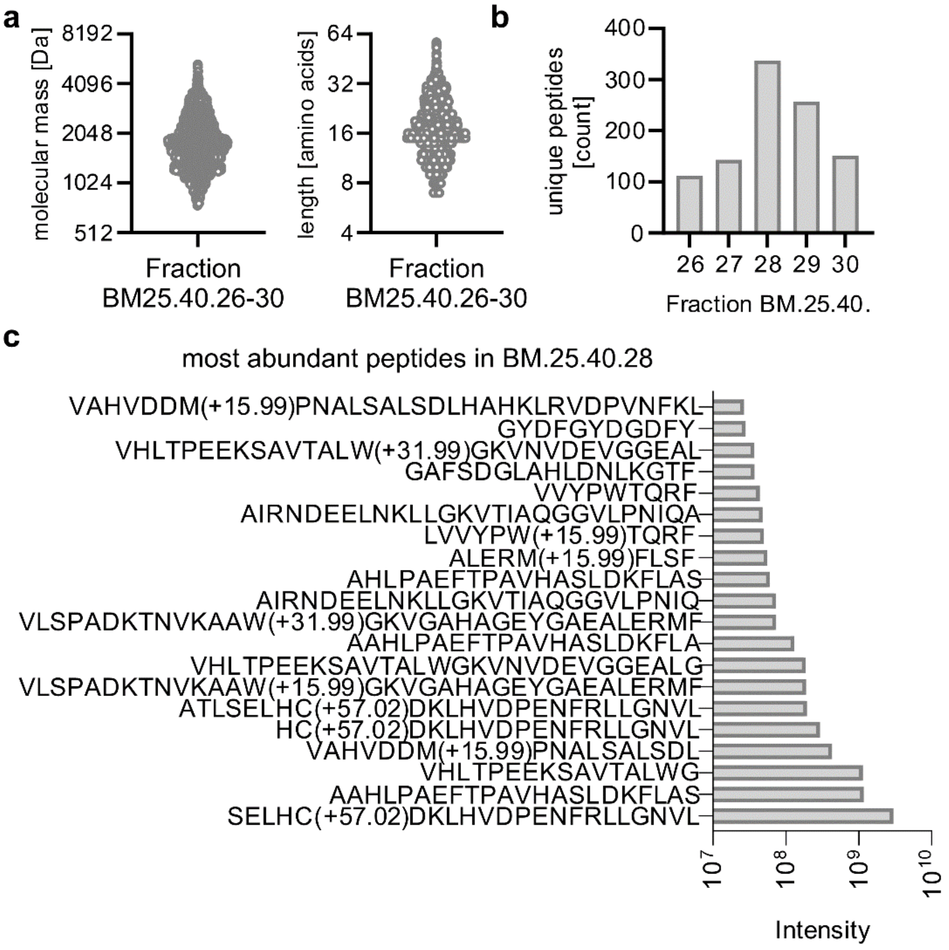

Supplementary Fig. 1 Identification of HBA1(111-132) in the bone marrow library. **a** Molecular mass (left panel) and amino acid length (right panel) distribution of all identified peptides in BM.25.40.26-30. **b** Number of unique peptide sequences identified in BM.25.40.26-30. **c** Intensity of the top 20 identified peptides in fraction 25.40.28 of the bone marrow library as analyzed by mass spectrometry.

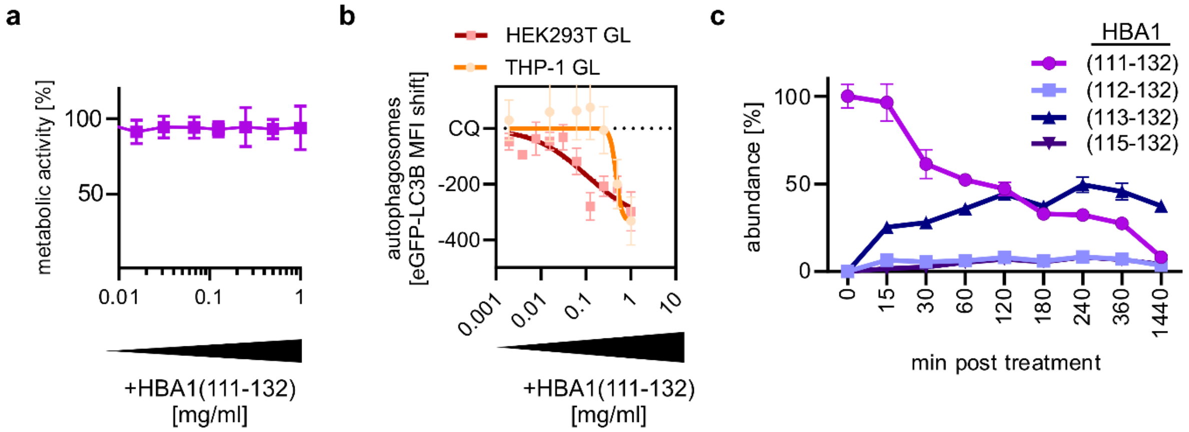

Supplementary Fig. 2 Characterization of chemically synthesized HBA1(111-132). **a** Metabolic activity was assessed after treatment of HeLa GL cells with HBA1(111-132) at concentrations ranging from 1 mg/ml to 7.8 µg/ml for 4 h using 3-(4,5-Dimethylthiazol-2-yl)-2,5-diphenyltetrazoliumbromide (MTT). **b** Quantification of autophagosome levels in HEK293T and THP-1 stably expressing GFP-LC3B reporter cells (HEK293T GL, THP-1 GL) 4 h post treatment with HBA1(111-132) (1 mg/ml - 1.9 µg/ml) by flow cytometry. Bars represent mean of n=3 ±SEM. **c** Intensity of indicated HBA1 fragment after incubation of HBA1(111-132) in human plasma. The y-axis represents the intensity of respective fragment in relation to the total intensity of all fragments at indicated time point (x-axis).

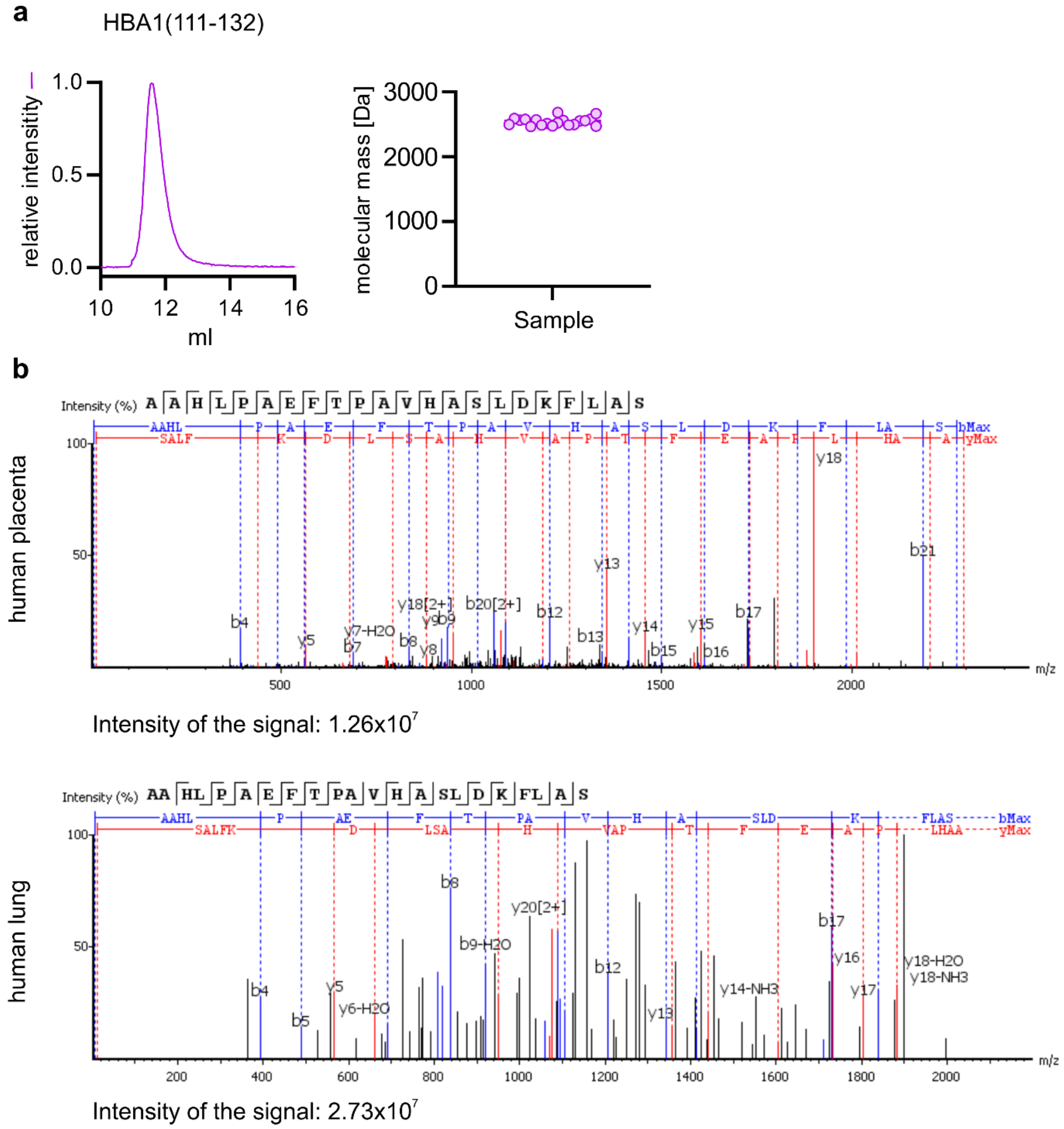

Supplementary Fig. 3 Characterization HBA1(111-132) in solution and detection in human tissues. **a** HBA1(111-132) exists in a monomeric state in solution. 5 mg/ml of HBA1(111-132) solved in PBS analyzed via multi-angle light scattering (MALS). The normalized light scattering intensity (left panel) is plotted against the retention volume. Molar masses calculated from experimental scattering (right panel), n=19. **b** Exemplary spectra of the mass spectrometry analysis of a human placenta and lung library. In blue and red are assigned amino acids. The intensity of the signal is indicated.

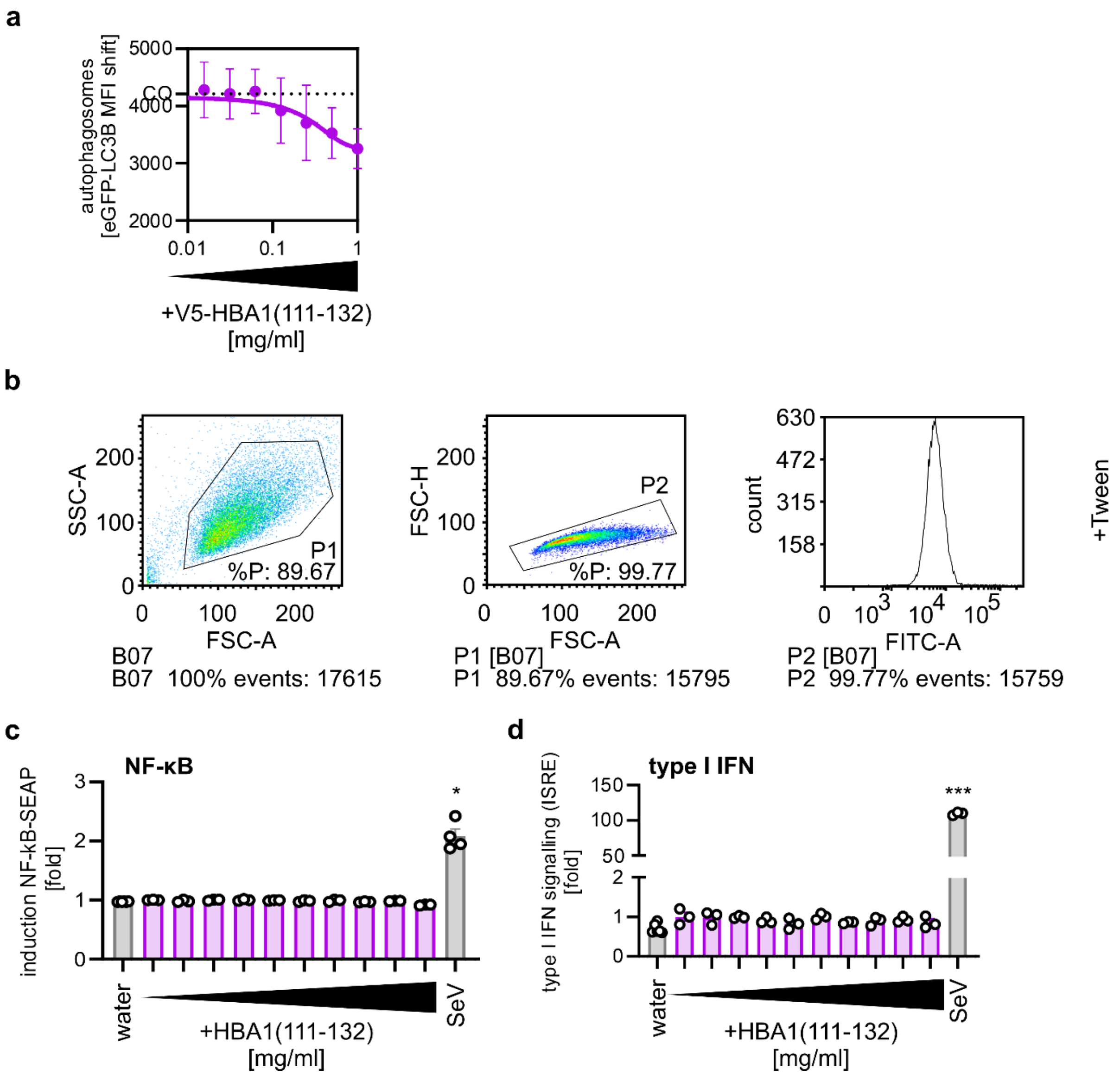

Supplementary Fig. 4 HBA1(111-132) exerts its autophagy inhibiting effect from the cell surface without causing inflammation. **a** Autophagosome levels in HeLa reporter cells stably expressing GFP-LC3B (HeLa GL) as assessed by flow cytometry 4 h post treatment using various concentrations (1mg/ml – 7.8 µg/ml) of V5 tagged HBA1(111-132). **b** Exemplary gating strategy for the localization analysis of V5-HBA1(111-132) in Fig 4c. Expected living cell population is gated in forward scatter (FSC)-A and side scatter (SSC)-A (left panel). Single cells were selected via FSC-H and FSC-A gating (middle panel). Fluorescence of V5-antibody is assessed in the FITC channel. **c** A549-Dual reporter cells were treated with HBA1(111-132) (1 mg/ml - 2 µg/ml). NF-κB driven secreted alkaline phosphatase expression and IFN stimulated response elements driven luciferase expression was quantified 24 h post treatment and normalized to water. SeV (Sendai Virus). Bars represent mean of n=3 ±SEM. Student's t-test with Welch correction. *, $p < 0.05$; ***, $p < 0.001$.

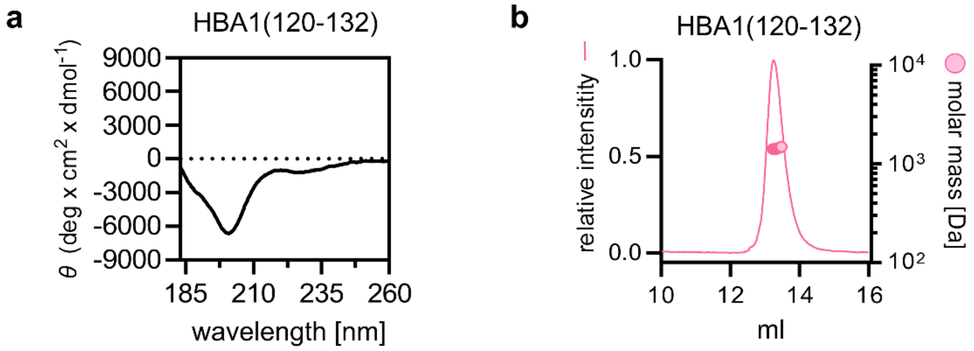

c

	aliphatic index	GRAVY	theoretical pI
120-PAVHASLDKFLAS-132	105.38	0.477	7.17
1-SALADKSLAPVHF-13	105.38	0.477	6.46
1-FSLKDLVASAAPH-13	105.38	0.477	6.74
120-PAVHASLAKFLAS-132	113.08	0.885	9.18
120-PAVHASLDAFLAS-132	113.08	0.915	5.08
120-PAVHASLAAFLAS-132	120.77	1.323	7.17
120-PAVHAALDKFLAS-132	113.08	0.677	7.17
120-PAVHASLDKFLAA-132	113.08	0.677	7.17
120-PAVHAALDKFLAA-132	120.77	0.877	7.17

Supplementary Fig. 5 Molecular properties of HBA1(111-132) variants. **a** Secondary structure of HBA1(120-132) was determined using circular dichroism spectrum in 10 mM PBS pH = 7.4 at 25 °C. **b** 5 mg/ml of HBA1(111-132) solved in PBS analyzed via multi-angle light scattering (MALS). The normalized light scattering intensity (left y-axis, line) is plotted against the retention volume. The right y-axis (dots) displays the molar masses calculated from experimental scattering. **c** Table with calculated properties (aliphatic index, GRAVY (Grand average of hydropathicity) and pI (theoretical isoelectric point)) of HBA1(120-132) and variants by expasy [87].

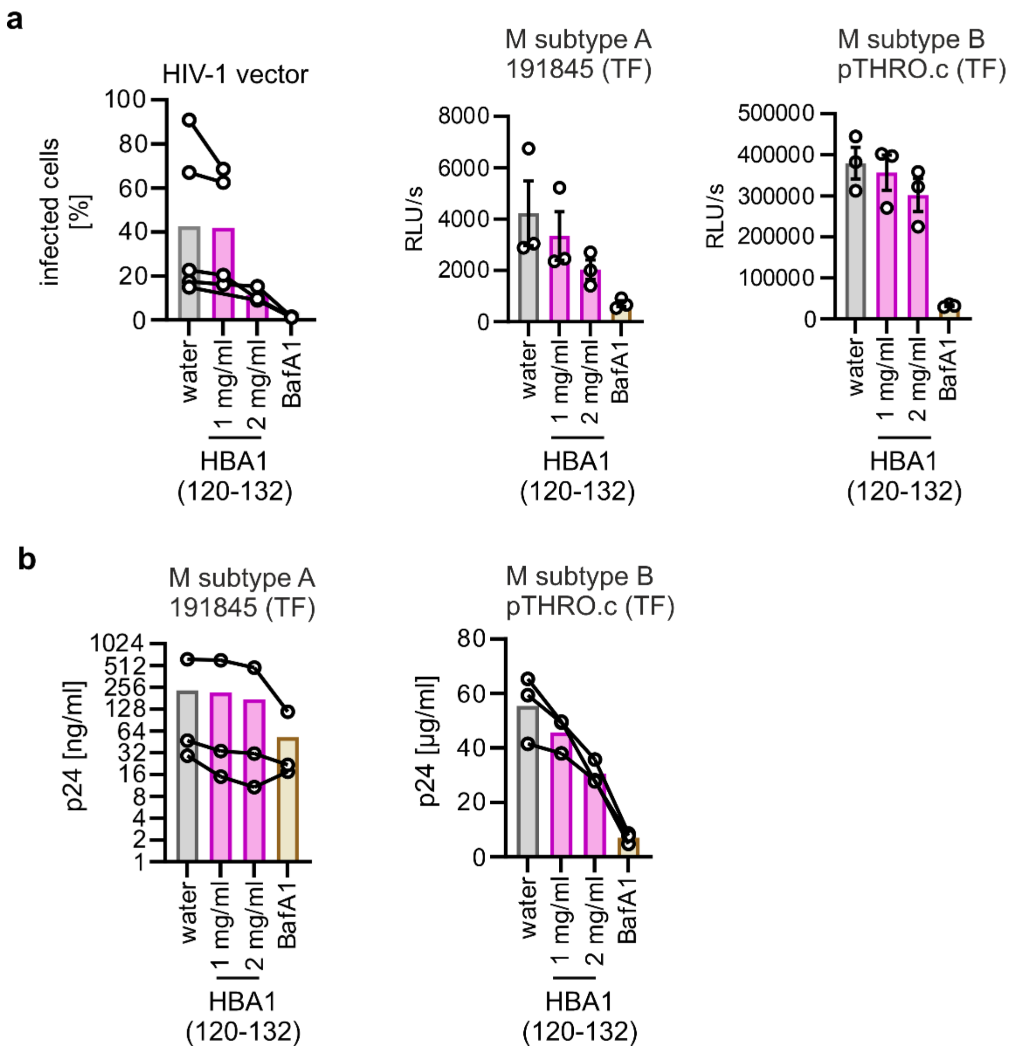

Supplementary Fig. 6 Anti-viral effect of HBA1(120-132) against HIV-1. **a** Raw values of Fig. 6b and c for indicated transmitted founder HIV-1 strain. Infected TZM-bl cells were quantified by GFP fluorescence using flow cytometry. Lines represent individual experiments, n=3-5 ±SEM. Relative light units per second (RLU/s) were recorded over a time period of 0.1 s. BafA1 (Bafilomycin A1, 250 nM). Bars represent the mean of n=3 ±SEM. **b** Raw values of the ELISA in Fig. 6d. Individual replicates are outlined.

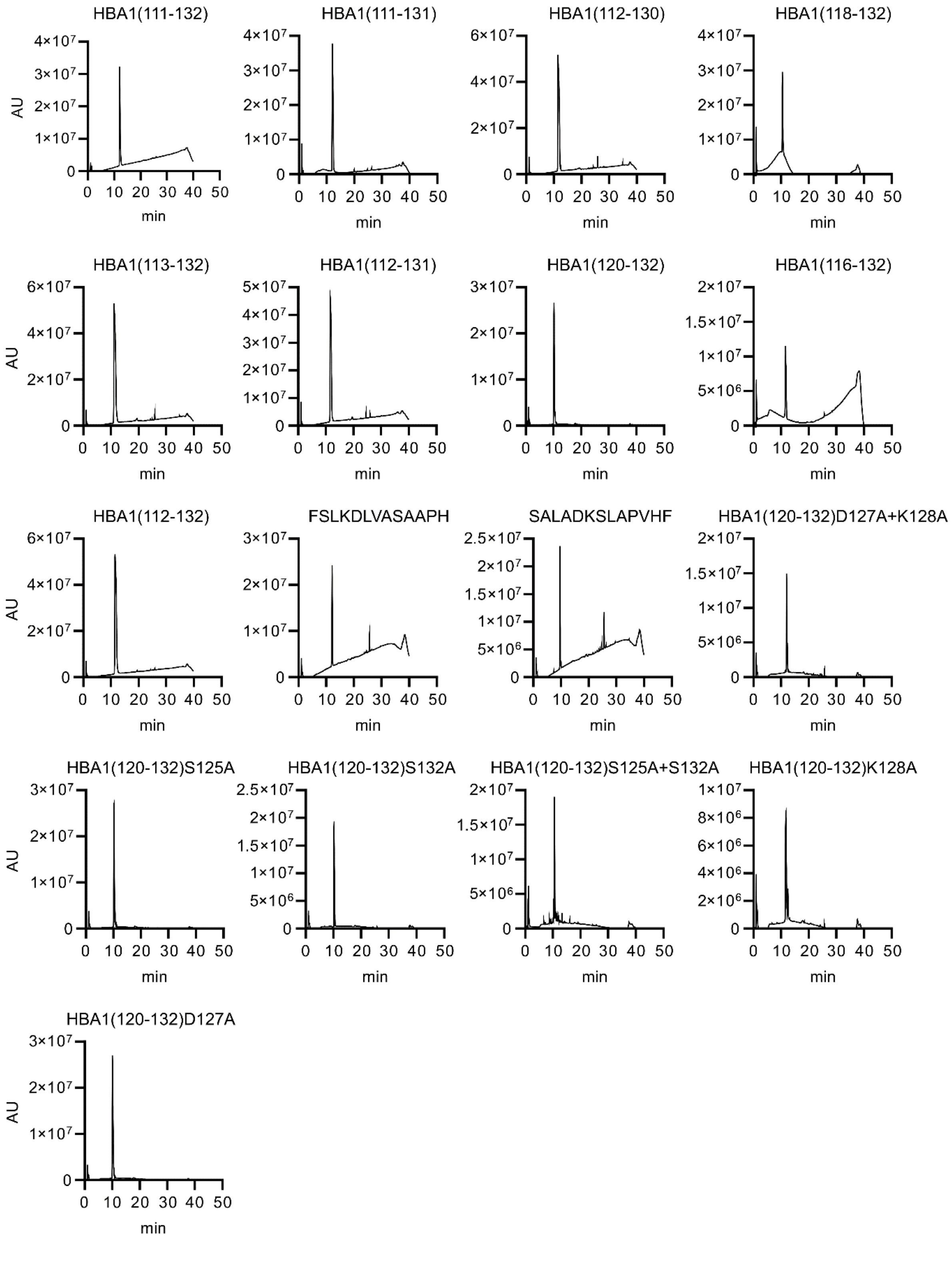

Supplementary Fig. 7 HPLC chromatogram of synthesized peptides. The retention time is shown on the x-axis and the absorbance at 214 nm is displayed on the y-axis. AU, absorption unit.
