# Supplementary material for: A naturally occurring 22-amino acid fragment of human hemoglobin A inhibits autophagy and HIV-1
Source: Cell Mol Life Sci. 2024 Sep 17;81(1):409. doi: 10.1007/s00018-024-05447-1 (PMC11408460; doi:10.1007/s00018-024-05447-1)
Supplement: Supplementary file 3 — Supplementary file3 (PDF 1341 KB) [file 18_2024_5447_MOESM3_ESM.pdf]

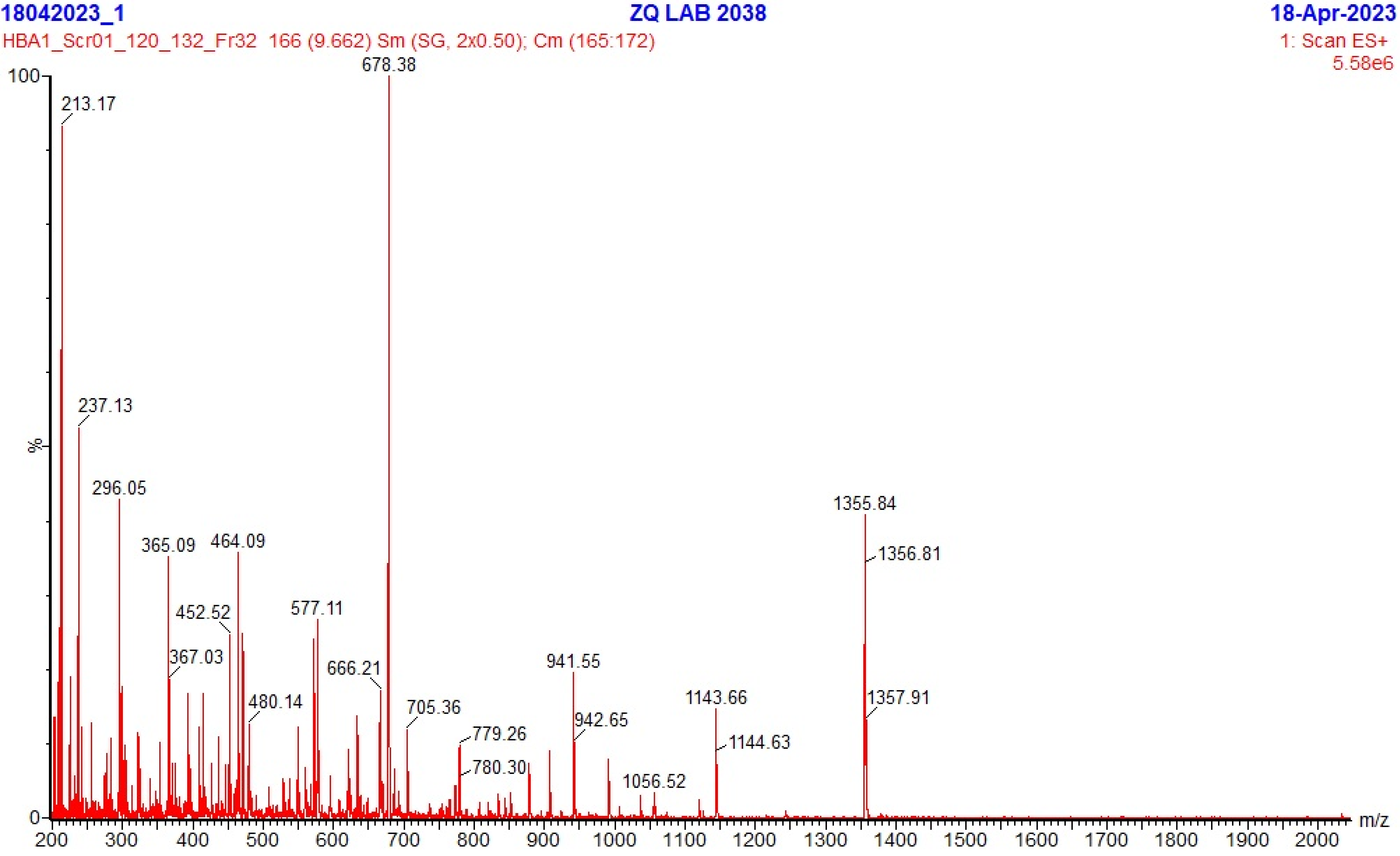
18042023_1
ZQ LAB 2038
18-Apr-2023
HBA1_Scr01_120_132_Fr32 166 (9.662) Sm (SG, 2x0.50); Cm (165:172)
1: Scan ES+
5.58e6
678.38
213.17
237.13
296.05
365.09
464.09
452.52
577.11
367.03
666.21
480.14
705.36
941.55
942.65
779.26
780.30
1056.52
1143.66
1144.63
1355.84
1356.81
1357.91
100
%
0
200
300
400
500
600
700
800
900
1000
1100
1200
1300
1400
1500
1600
1700
1800
1900
2000
m/z

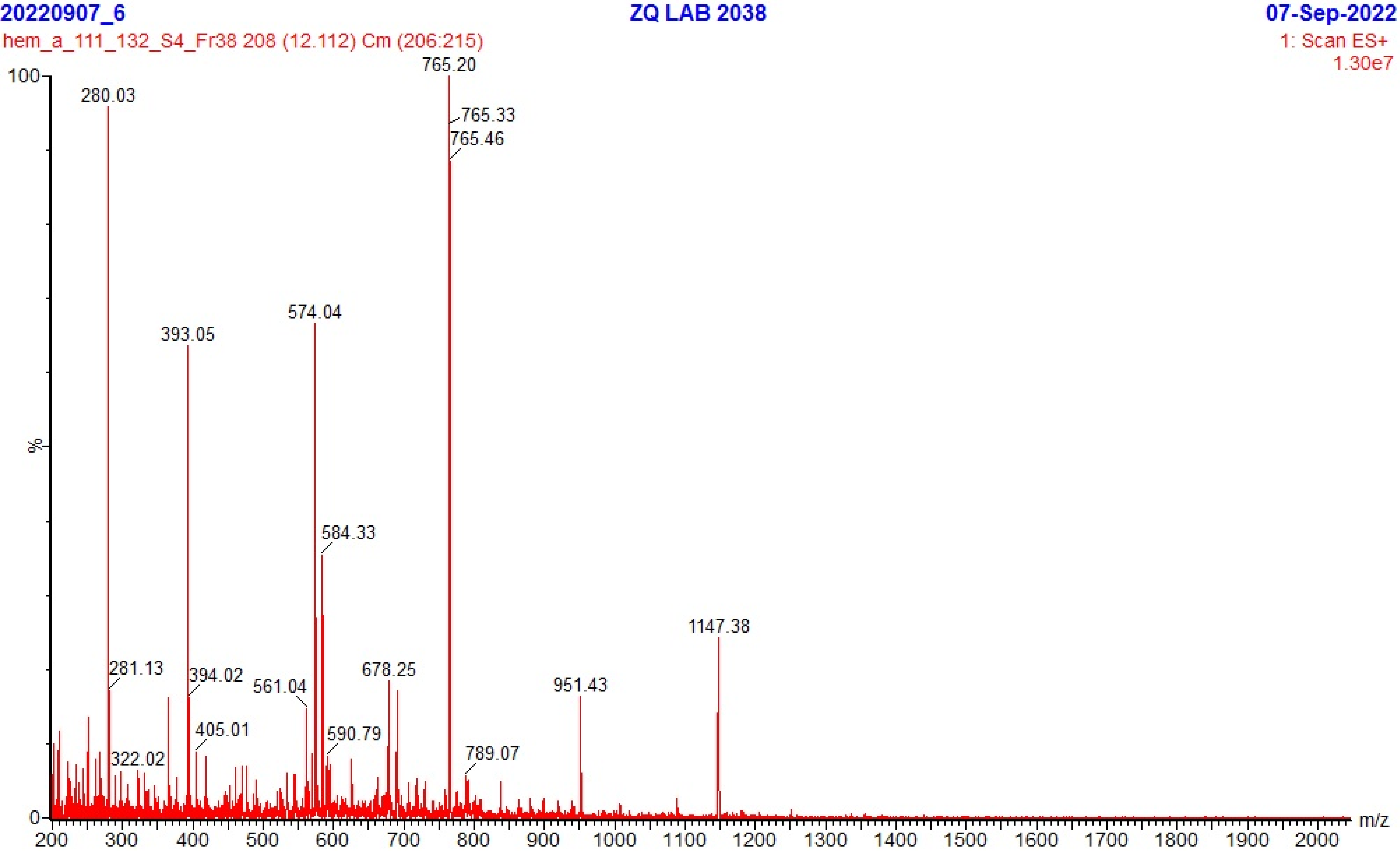
20220907_6
ZQ LAB 2038
07-Sep-2022
hem_a_111_132_S4_Fr38 208 (12.112) Cm (206:215)
1: Scan ES+
1.30e7
765.20
765.33
765.46
280.03
574.04
393.05
584.33
1147.38
281.13
394.02
561.04
678.25
951.43
405.01
590.79
322.02
789.07
%
100
0
m/z
200
300
400
500
600
700
800
900
1000
1100
1200
1300
1400
1500
1600
1700
1800
1900
2000

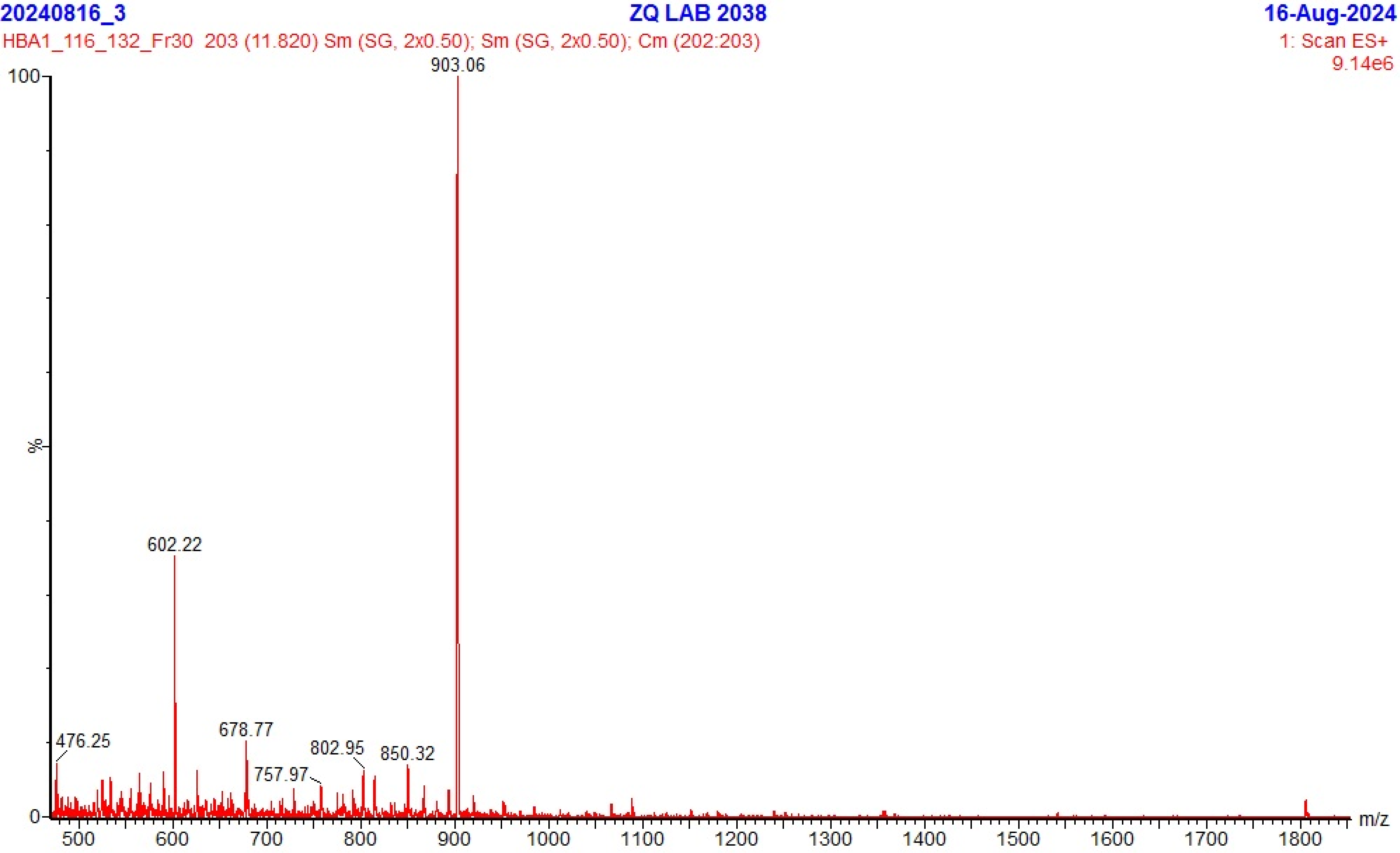
20240816_3
ZQ LAB 2038
16-Aug-2024
HBA1_116_132_Fr30 203 (11.820) Sm (SG, 2x0.50); Sm (SG, 2x0.50); Cm (202:203)
1: Scan ES+
9.14e6
903.06
602.22
476.25
678.77
757.97
802.95
850.32
100
%
0
m/z
500
600
700
800
900
1000
1100
1200
1300
1400
1500
1600
1700
1800

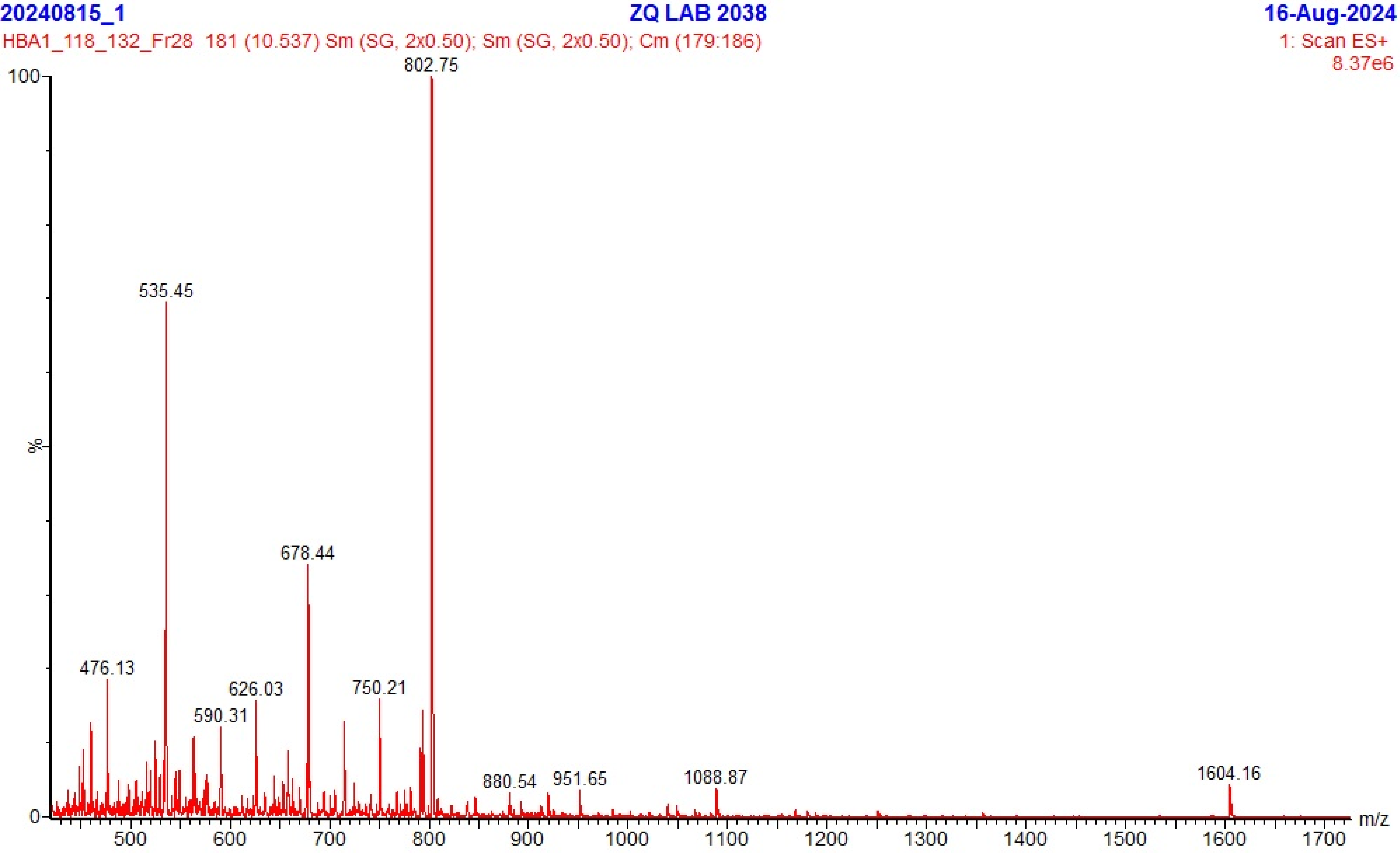
20240815_1
ZQ LAB 2038
16-Aug-2024
HBA1_118_132_Fr28 181 (10.537) Sm (SG, 2x0.50); Sm (SG, 2x0.50); Cm (179:186)
1: Scan ES+
8.37e6
802.75
535.45
678.44
476.13
626.03
750.21
590.31
880.54
951.65
1088.87
1604.16
100
%
0
m/z
500
600
700
800
900
1000
1100
1200
1300
1400
1500
1600
1700

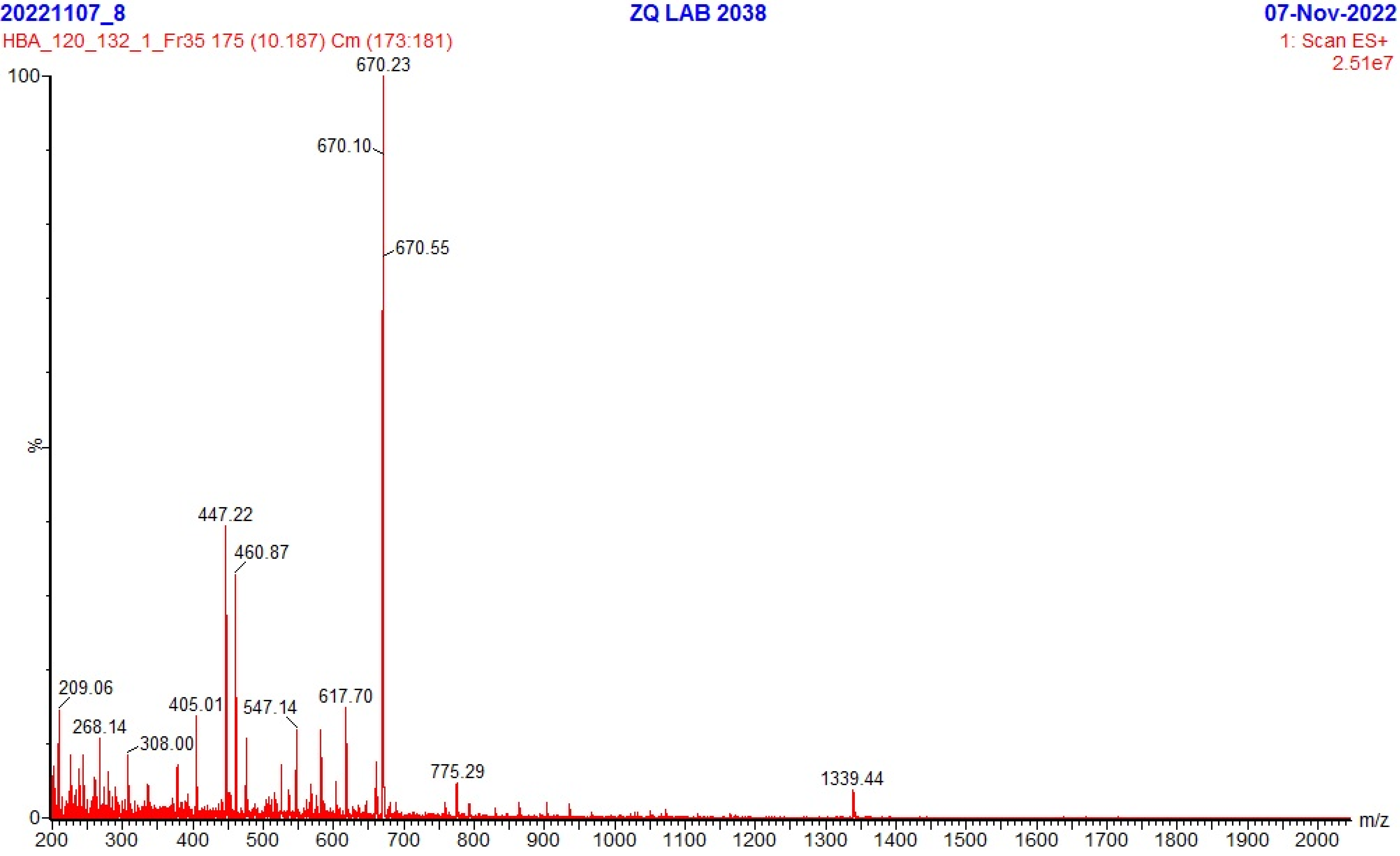
20221107_8
ZQ LAB 2038
07-Nov-2022
HBA_120_132_1_Fr35 175 (10.187) Cm (173:181)
1: Scan ES+
2.51e7
670.23
670.10
670.55
447.22
460.87
209.06
405.01
547.14
617.70
268.14
308.00
775.29
1339.44
100
%
0
200
300
400
500
600
700
800
900
1000
1100
1200
1300
1400
1500
1600
1700
1800
1900
2000
m/z

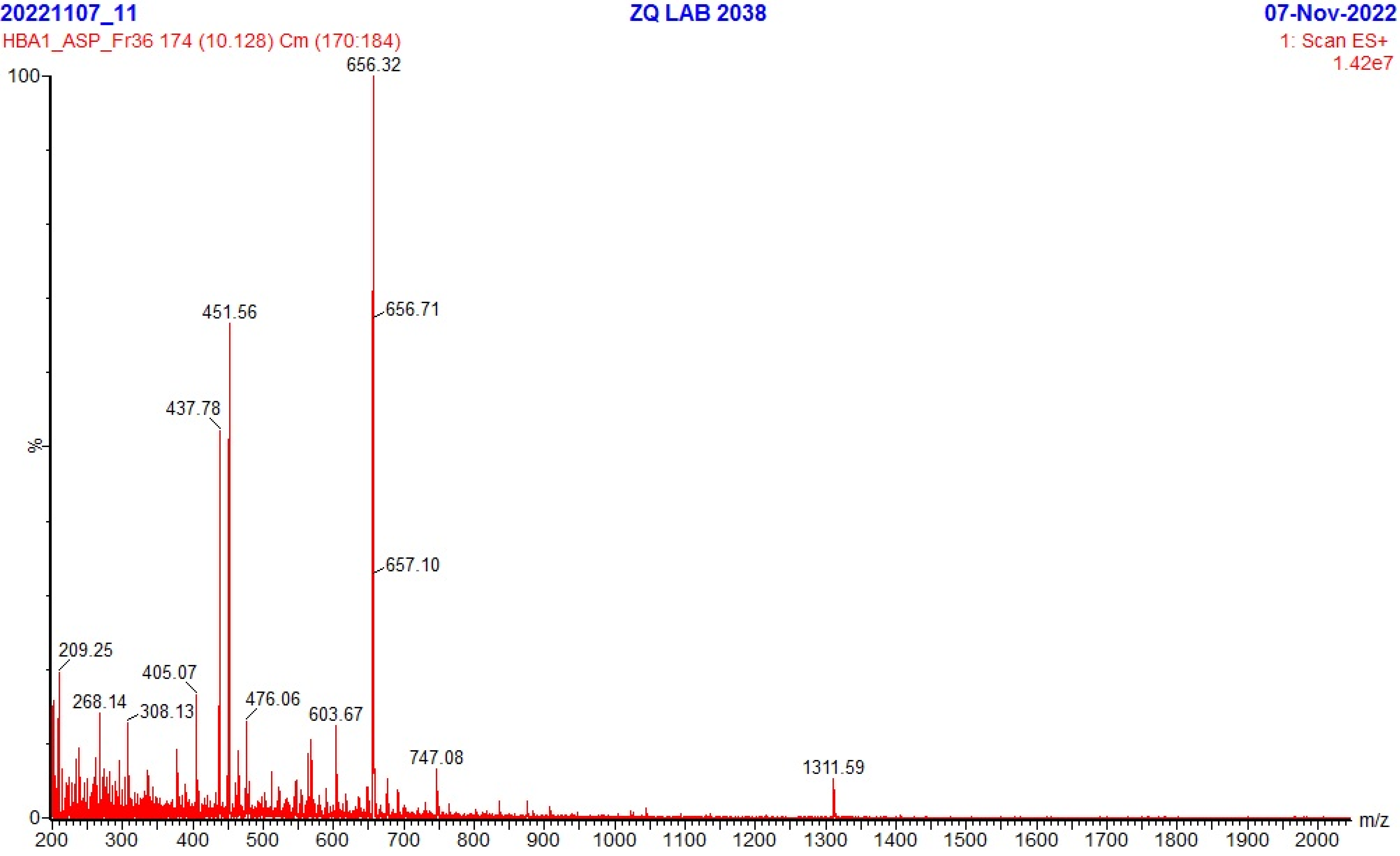
20221107_11
ZQ LAB 2038
07-Nov-2022
HBA1_ASP_Fr36 174 (10.128) Cm (170:184)
1: Scan ES+
1.42e7
656.32
656.71
657.10
451.56
437.78
209.25
405.07
268.14
308.13
476.06
603.67
747.08
1311.59
100
%
0
200
300
400
500
600
700
800
900
1000
1100
1200
1300
1400
1500
1600
1700
1800
1900
2000
m/z

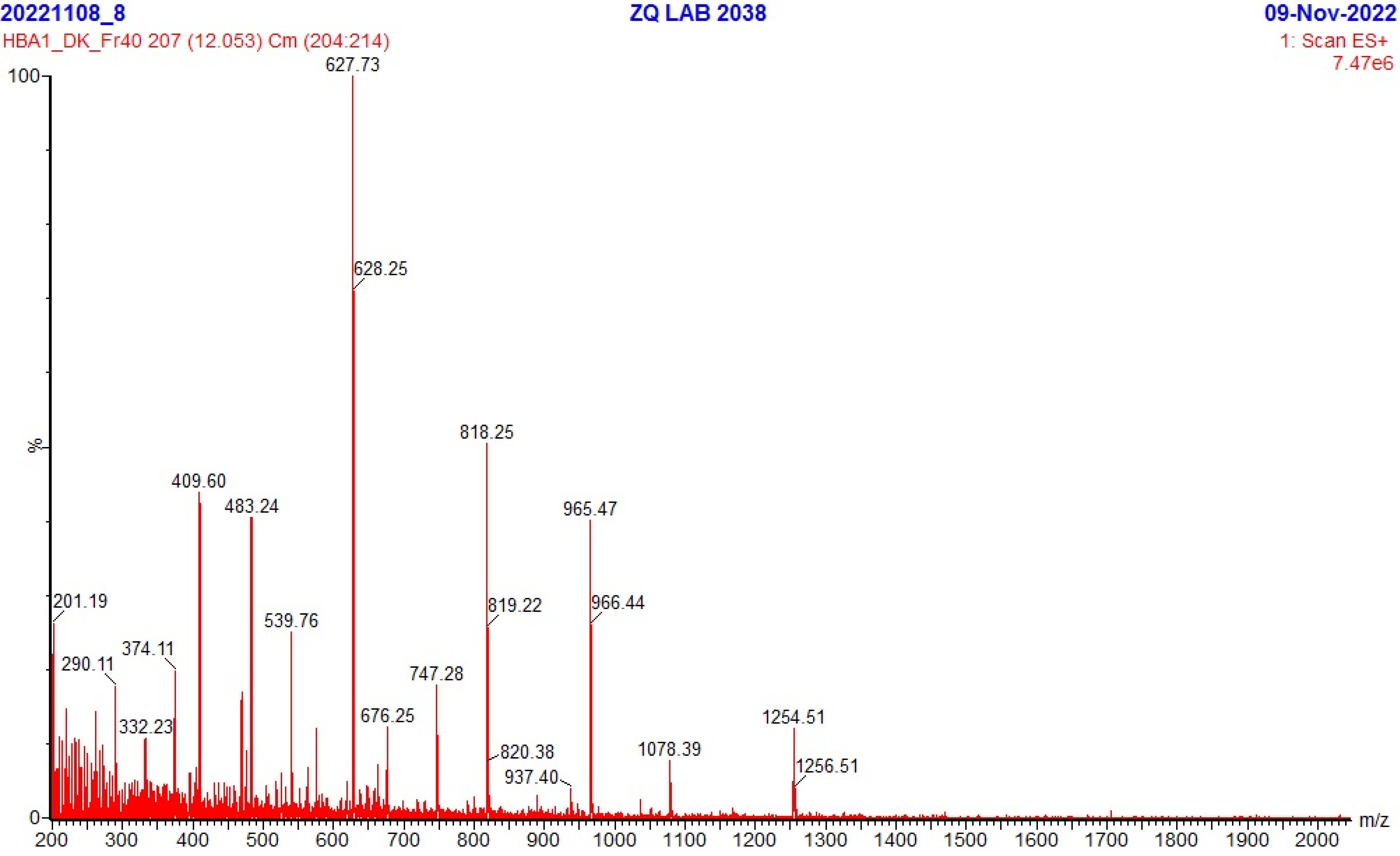

20221108_8
ZQ LAB 2038
09-Nov-2022
HBA1_DK_Fr40 207 (12.053) Cm (204:214)
1: Scan ES+
7.47e6
100
%
0
627.73
628.25
818.25
409.60
483.24
965.47
201.19
819.22
966.44
539.76
374.11
290.11
747.28
332.23
676.25
1254.51
1078.39
820.38
1256.51
937.40
200
300
400
500
600
700
800
900
1000
1100
1200
1300
1400
1500
1600
1700
1800
1900
2000
m/z

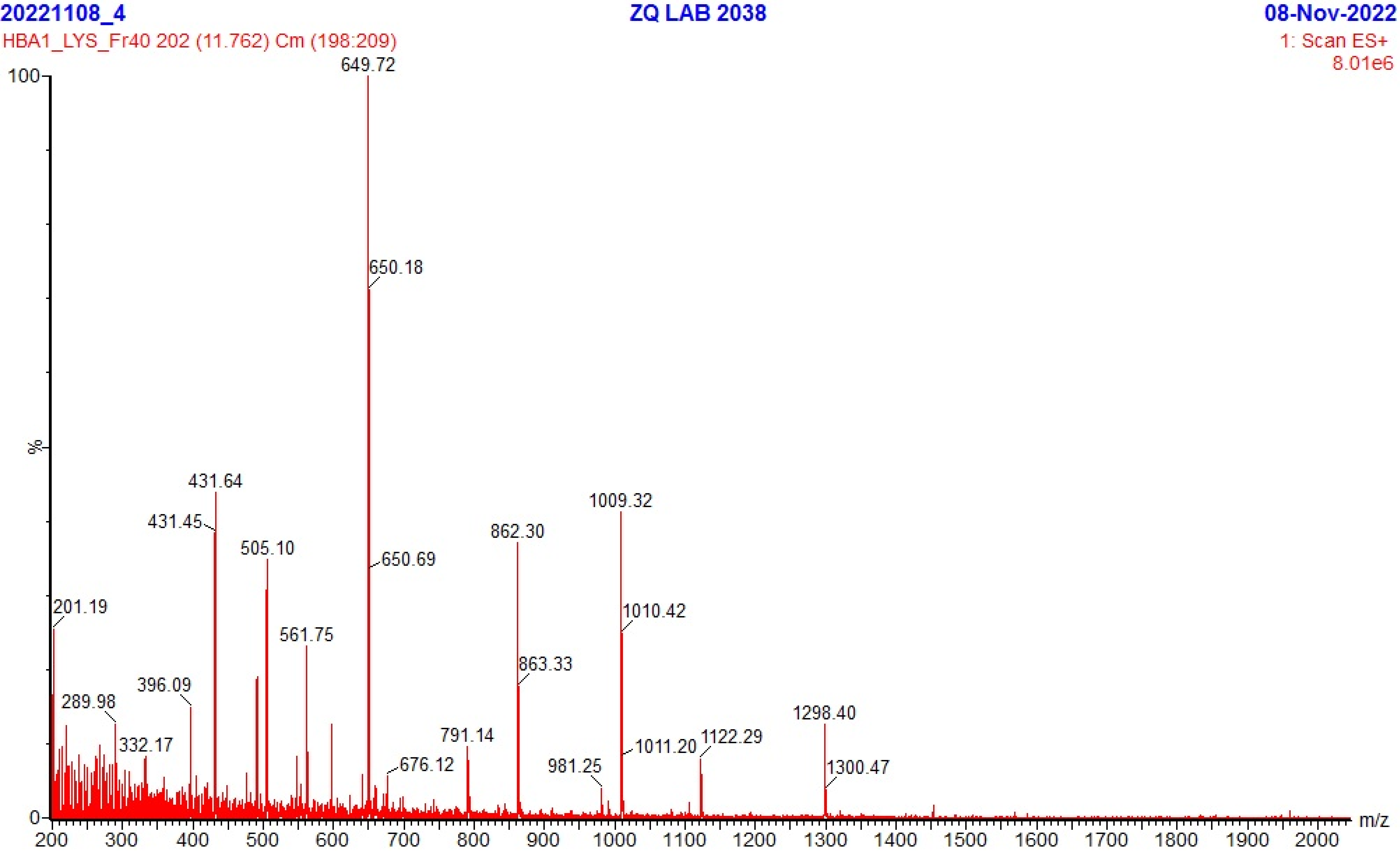

20221108_4
ZQ LAB 2038
08-Nov-2022
HBA1_LYS_Fr40 202 (11.762) Cm (198:209)
1: Scan ES+
8.01e6
100
%
0
649.72
650.18
650.69
431.64
431.45
505.10
561.75
201.19
289.98
332.17
396.09
676.12
791.14
862.30
863.33
981.25
1009.32
1010.42
1011.20
1122.29
1298.40
1300.47
200
300
400
500
600
700
800
900
1000
1100
1200
1300
1400
1500
1600
1700
1800
1900
2000
m/z

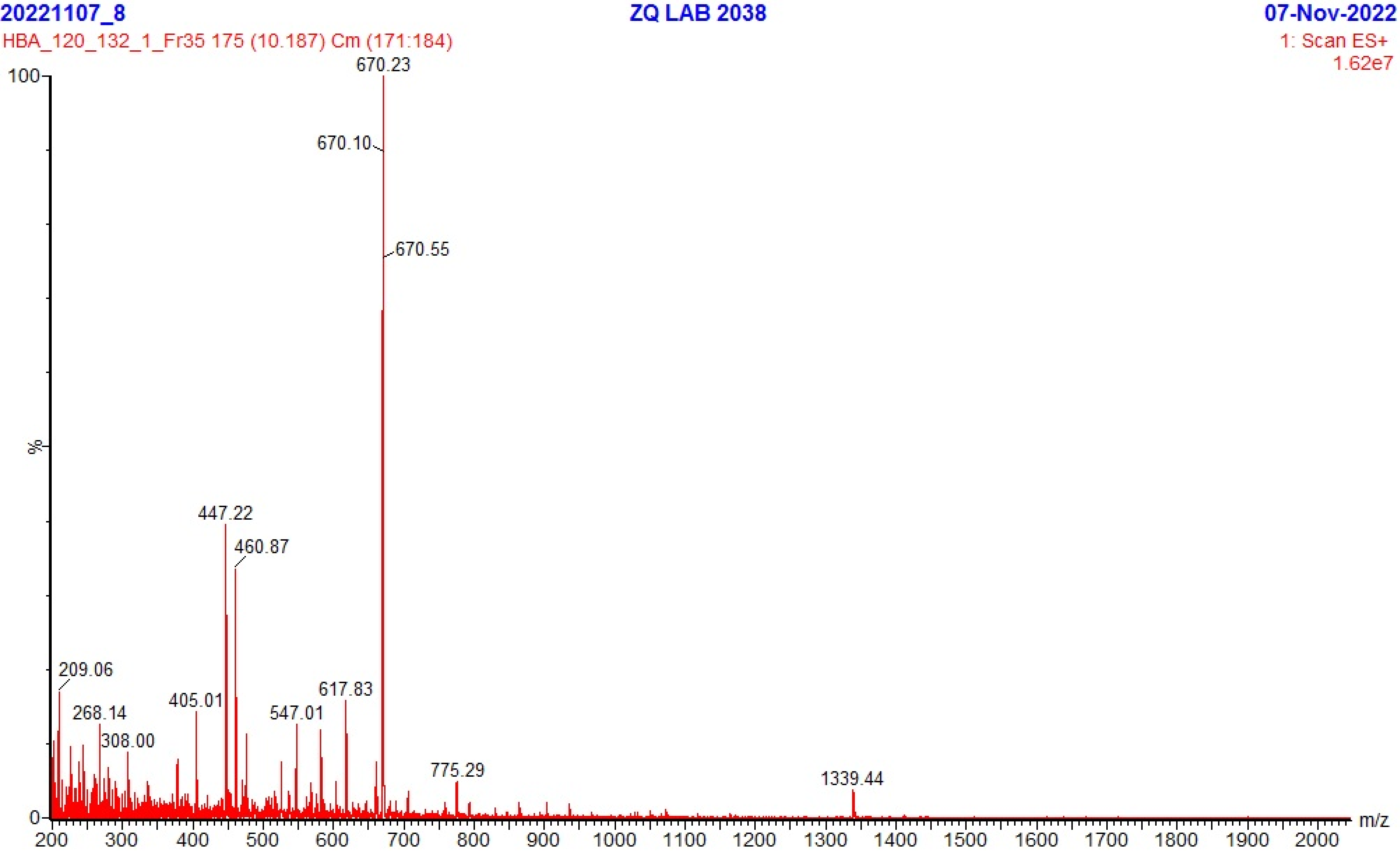

20221107_8
ZQ LAB 2038
07-Nov-2022
HBA_120_132_1_Fr35 175 (10.187) Cm (171:184)
1: Scan ES+
1.62e7
670.23
670.10
670.55
447.22
460.87
209.06
268.14
308.00
405.01
547.01
617.83
775.29
1339.44
100
%
0
200
300
400
500
600
700
800
900
1000
1100
1200
1300
1400
1500
1600
1700
1800
1900
2000
m/z

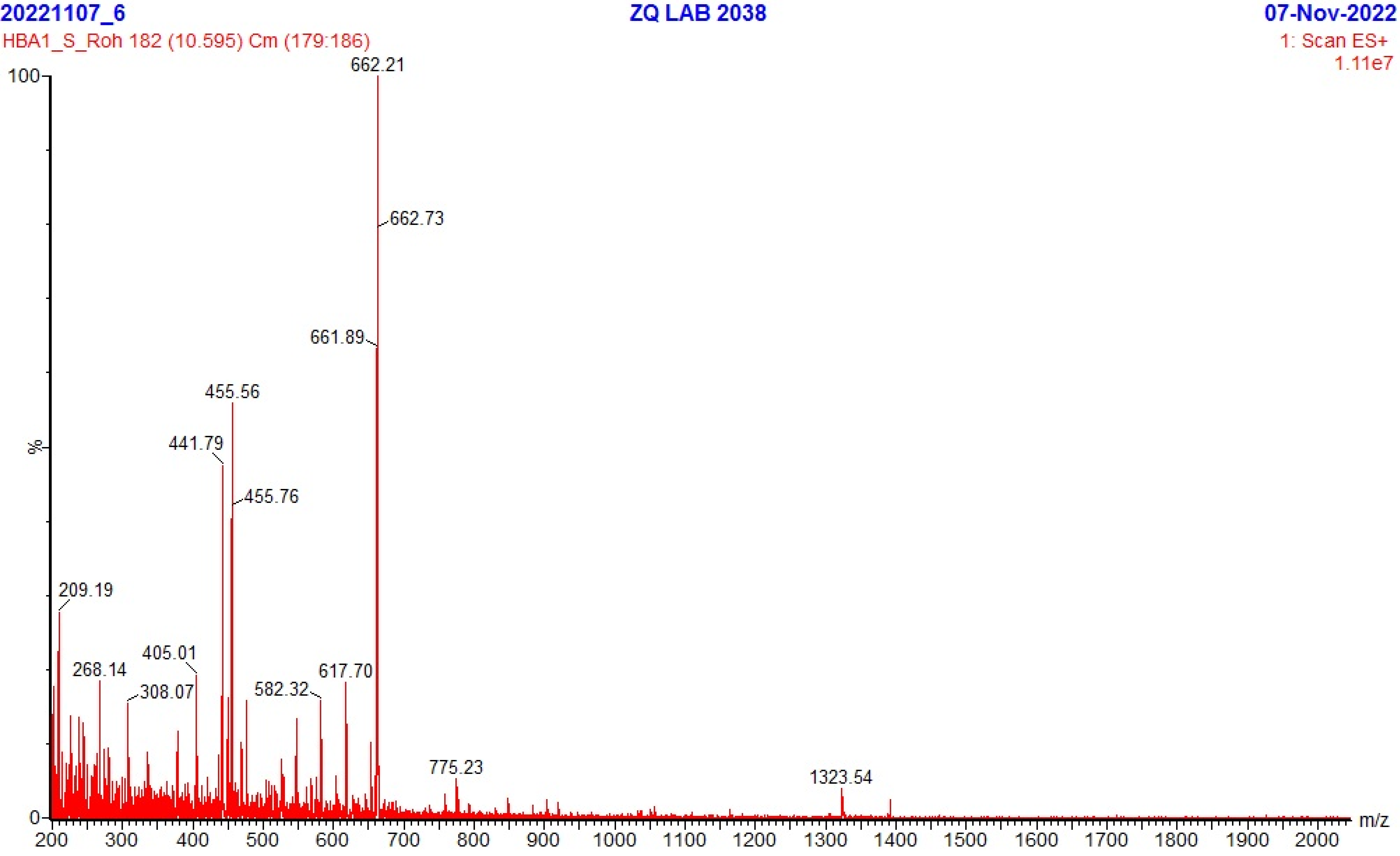

20221107_6
ZQ LAB 2038
07-Nov-2022
HBA1_S_Roh 182 (10.595) Cm (179:186)
1: Scan ES+
1.11e7
662.21
662.73
661.89
455.56
441.79
455.76
209.19
405.01
268.14
308.07
617.70
582.32
775.23
1323.54
100
%
0
200
300
400
500
600
700
800
900
1000
1100
1200
1300
1400
1500
1600
1700
1800
1900
2000
m/z

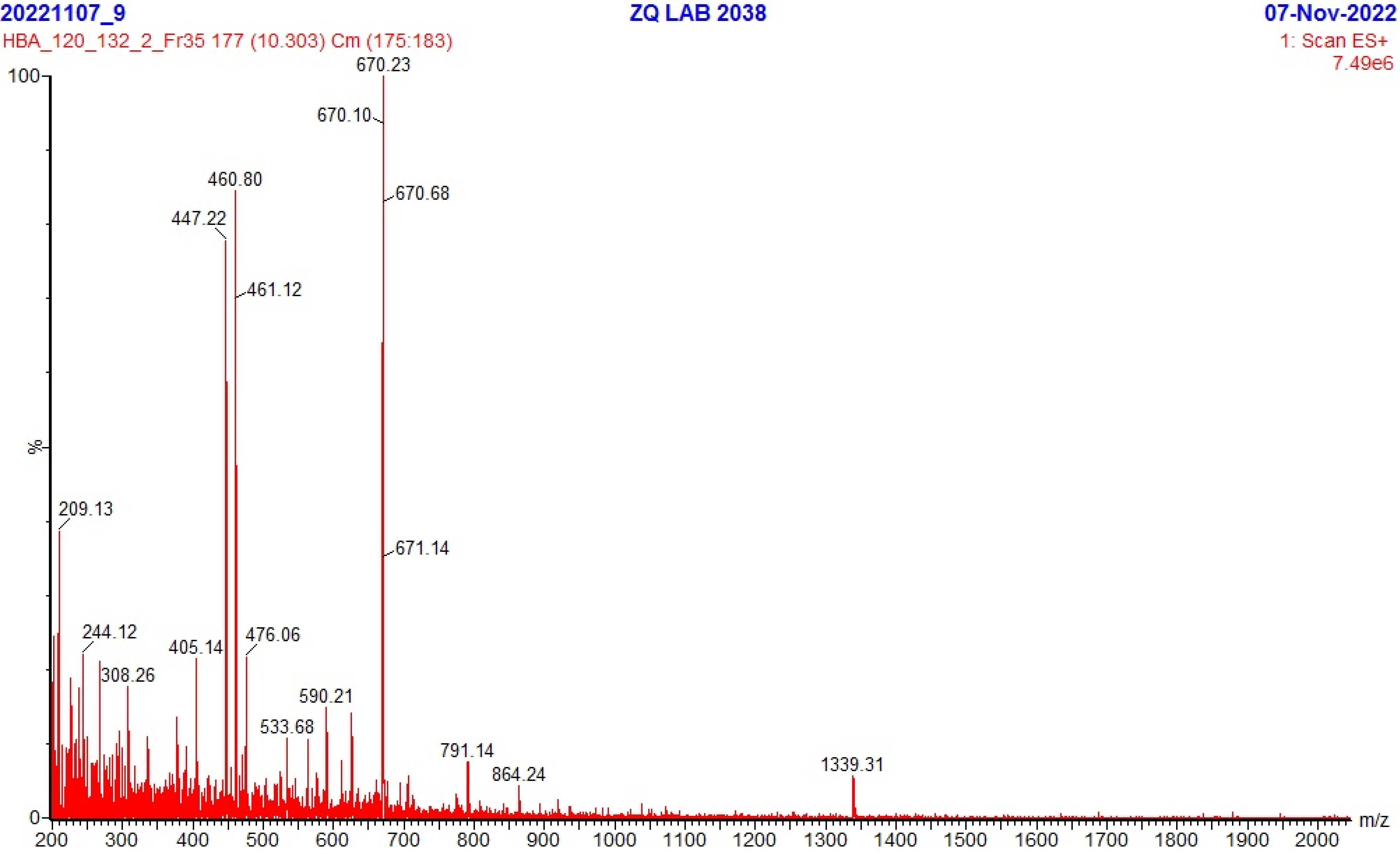

20221107_9
ZQ LAB 2038
07-Nov-2022
HBA_120_132_2_Fr35 177 (10.303) Cm (175:183)
1: Scan ES+
7.49e6
670.23
670.10
670.68
460.80
447.22
461.12
671.14
209.13
244.12
405.14
476.06
308.26
590.21
533.68
791.14
864.24
1339.31
100
%
0
200
300
400
500
600
700
800
900
1000
1100
1200
1300
1400
1500
1600
1700
1800
1900
2000
m/z

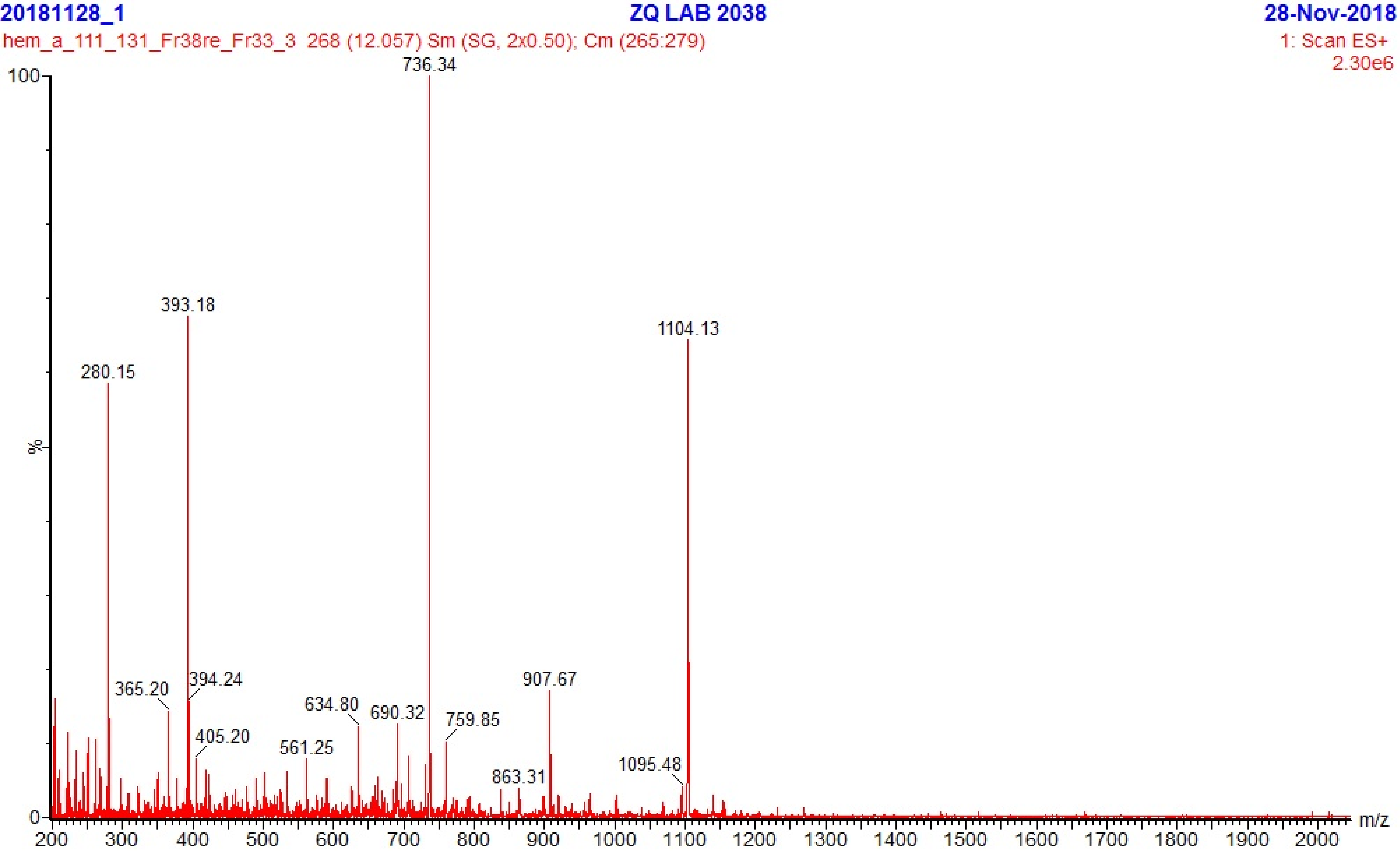
20181128_1
ZQ LAB 2038
28-Nov-2018
hem_a_111_131_Fr38re_Fr33_3 268 (12.057) Sm (SG, 2x0.50); Cm (265:279)
1: Scan ES+
2.30e6
736.34
393.18
280.15
1104.13
365.20
394.24
405.20
561.25
634.80
690.32
759.85
907.67
863.31
1095.48
100
%
0
m/z
200
300
400
500
600
700
800
900
1000
1100
1200
1300
1400
1500
1600
1700
1800
1900
2000

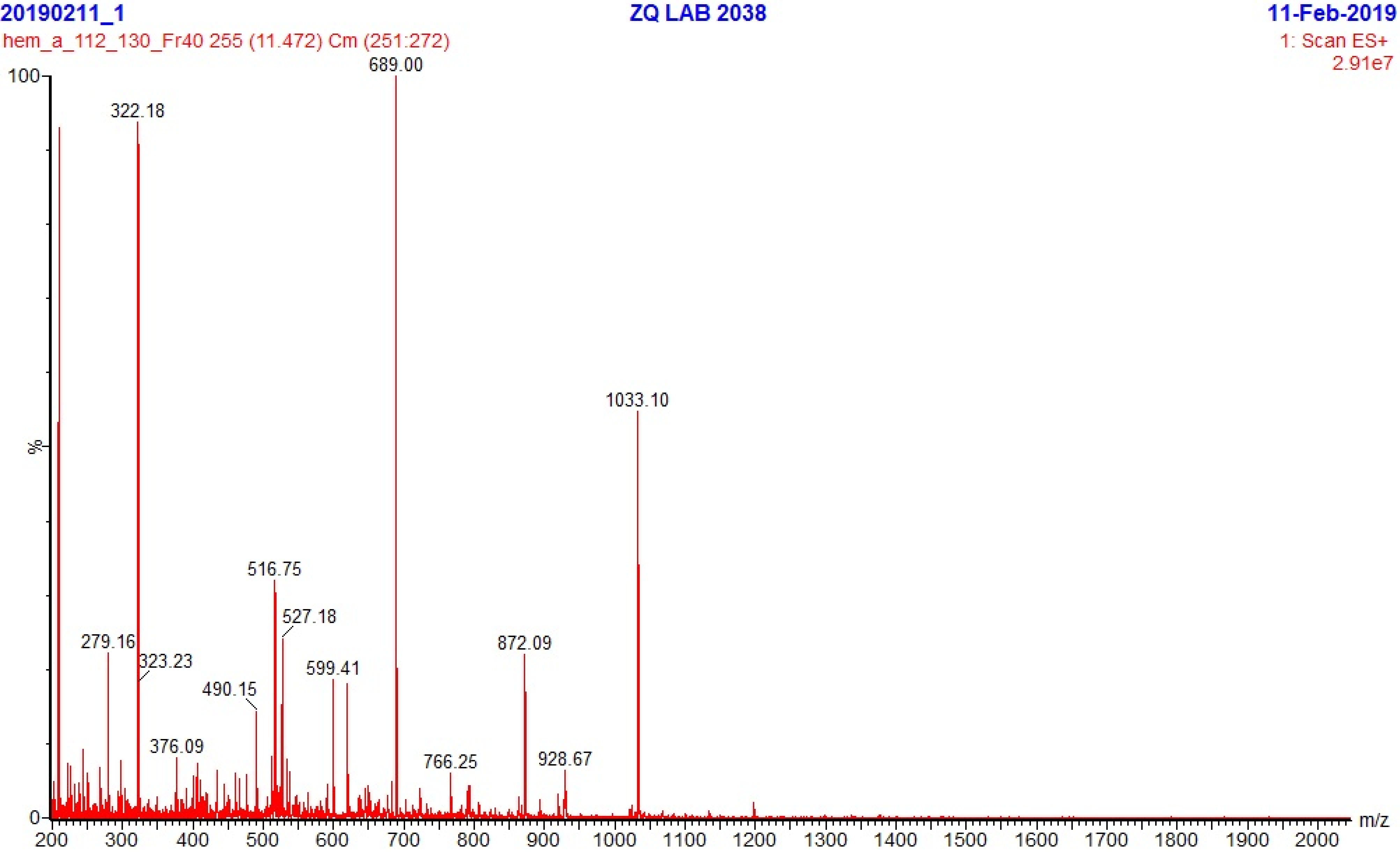
20190211_1
ZQ LAB 2038
11-Feb-2019
hem_a_112_130_Fr40 255 (11.472) Cm (251:272)
1: Scan ES+
2.91e7
689.00
322.18
1033.10
516.75
527.18
279.16
323.23
872.09
599.41
490.15
376.09
766.25
928.67
100
%
0
m/z
200
300
400
500
600
700
800
900
1000
1100
1200
1300
1400
1500
1600
1700
1800
1900
2000

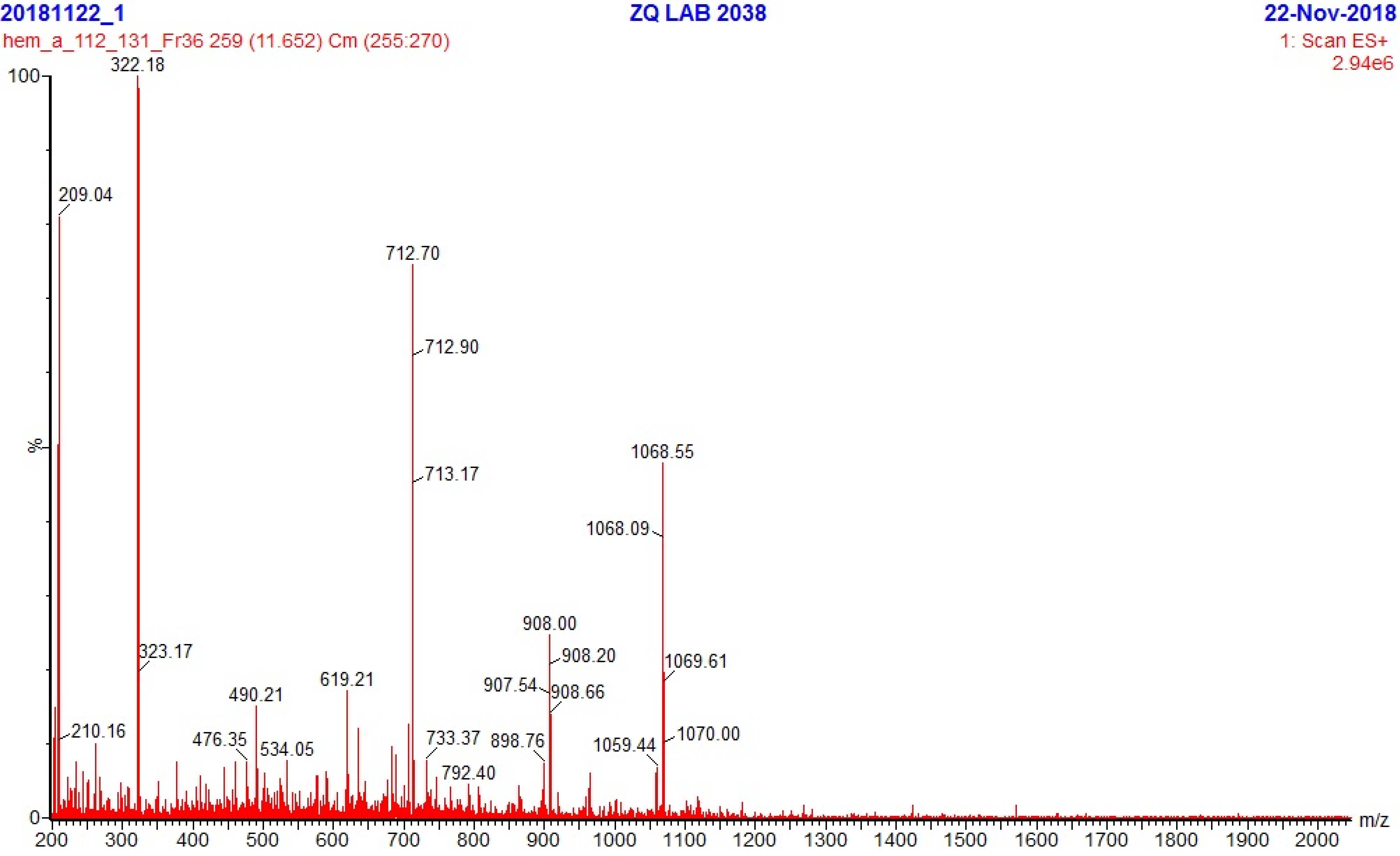
20181122_1
ZQ LAB 2038
22-Nov-2018
hem_a_112_131_Fr36 259 (11.652) Cm (255:270)
1: Scan ES+
2.94e6
322.18
209.04
712.70
712.90
713.17
1068.55
1068.09
908.00
908.20
907.54
908.66
1069.61
323.17
619.21
490.21
210.16
476.35
534.05
733.37
898.76
1059.44
1070.00
792.40
%
m/z

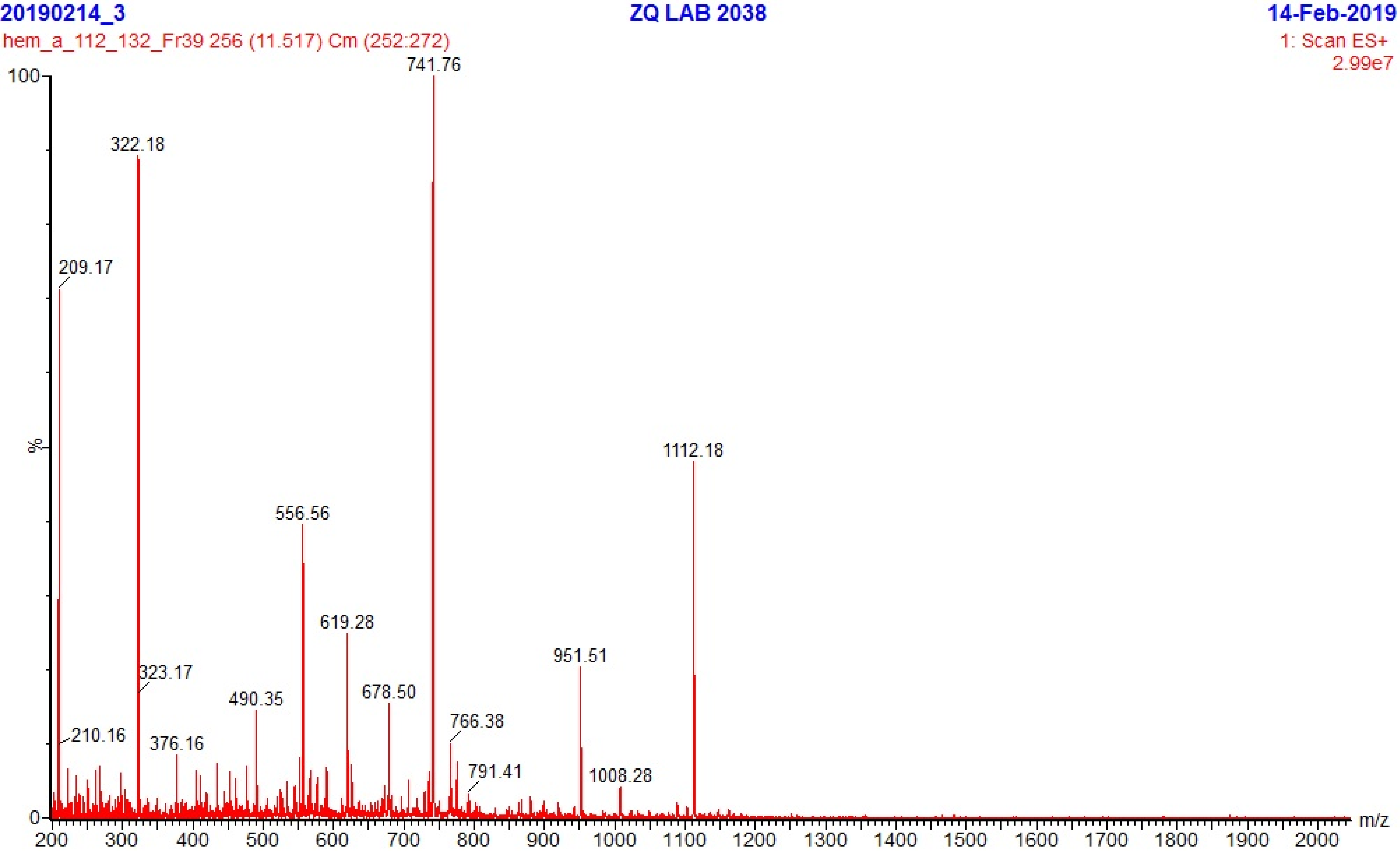

20190214_3
ZQ LAB 2038
14-Feb-2019
hem_a_112_132_Fr39 256 (11.517) Cm (252:272)
1: Scan ES+
2.99e7
741.76
322.18
209.17
1112.18
556.56
619.28
951.51
323.17
490.35
678.50
766.38
210.16
376.16
791.41
1008.28
%
100
0
m/z
200 300 400 500 600 700 800 900 1000 1100 1200 1300 1400 1500 1600 1700 1800 1900 2000

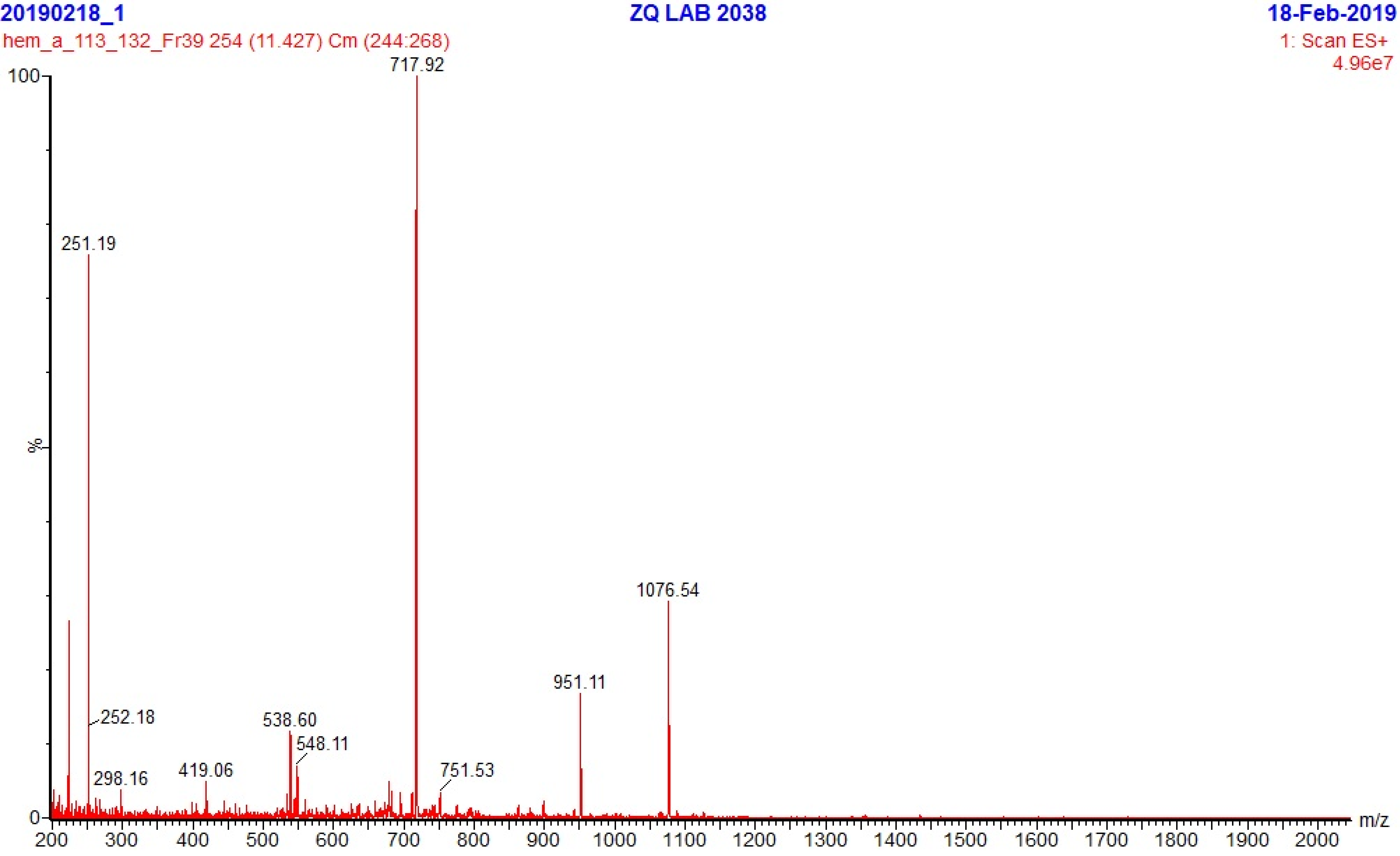

20190218_1
ZQ LAB 2038
18-Feb-2019
hem_a_113_132_Fr39 254 (11.427) Cm (244:268)
1: Scan ES+
4.96e7
717.92
251.19
1076.54
951.11
252.18
538.60
548.11
298.16
419.06
751.53
100
%
0
200
300
400
500
600
700
800
900
1000
1100
1200
1300
1400
1500
1600
1700
1800
1900
2000
m/z

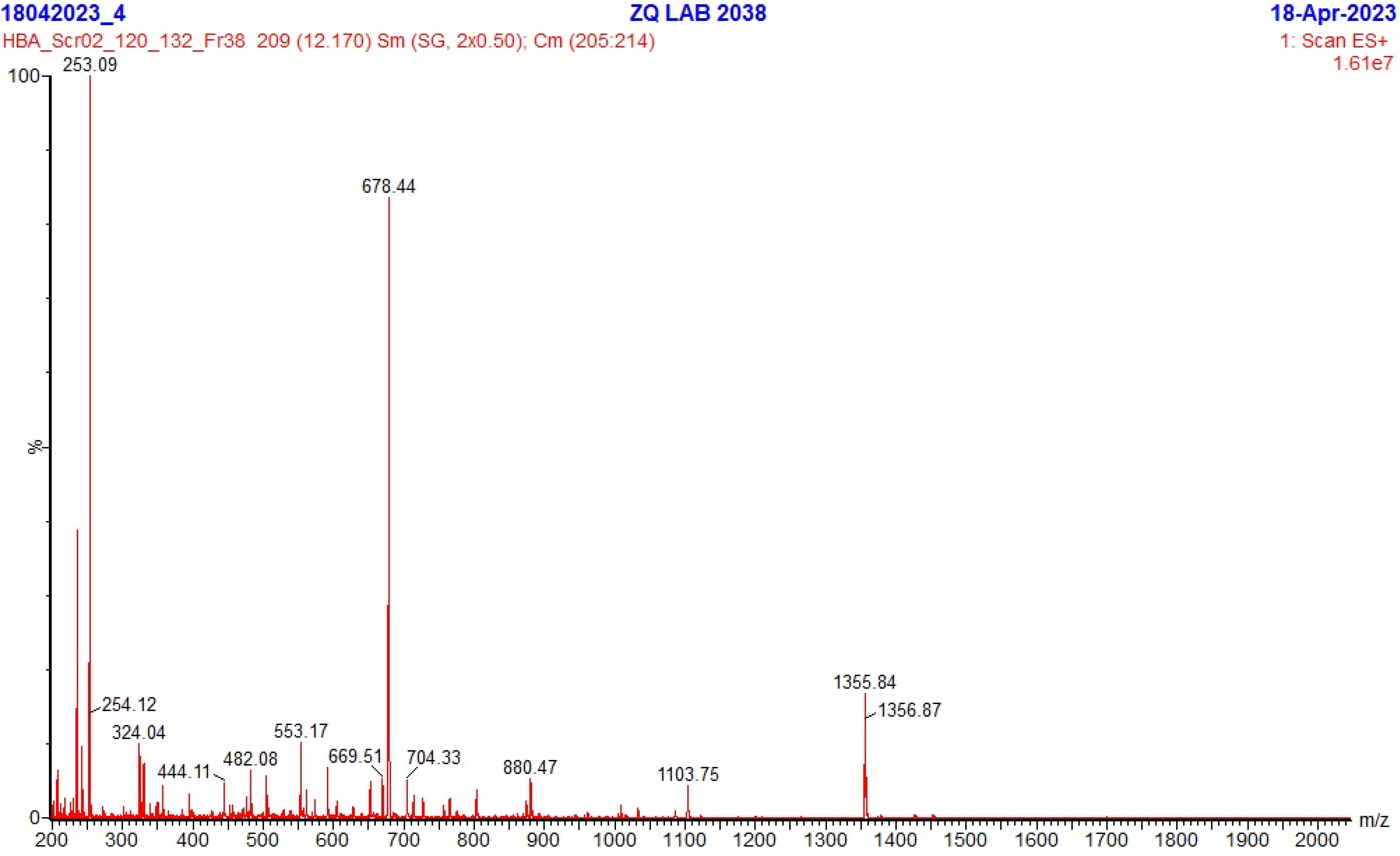
18042023_4
ZQ LAB 2038
18-Apr-2023
HBA_Scr02_120_132_Fr38 209 (12.170) Sm (SG, 2x0.50); Cm (205:214)
1: Scan ES+
1.61e7
100
%
0
253.09
678.44
254.12
324.04
444.11
482.08
553.17
669.51
704.33
880.47
1103.75
1355.84
1356.87
200
300
400
500
600
700
800
900
1000
1100
1200
1300
1400
1500
1600
1700
1800
1900
2000
m/z
